# Supplementary material for: Longitudinal imaging of therapeutic enzyme expression after gene therapy for Fabry disease using positron emission tomography and the radiotracer [18F]AGAL
Source: Mol Ther. 2024 Nov 19;33(9):4381–93. doi: 10.1016/j.ymthe.2024.11.021 (PMC12432858; doi:10.1016/j.ymthe.2024.11.021)
Supplement: Supplementary file 1 [file mmc1.pdf]

YMTHE, Volume *33*

Supplemental Information

Longitudinal imaging of therapeutic enzyme expression after gene therapy for Fabry disease using positron emission tomography and the radiotracer [^{18}F]AGAL

Charalambos Kaittanis, Tyler Teceno, Ashley Knight, Yoann Petibon, Phil Sandoval, Lawrence Cohen, Shin Hye Ahn, Anthony P. Belanger, Louise M. Clark, Quang-De Nguyen, Wanida Ruangsiriluk, Shreya Mukherji, Cristian C. Constantinescu, Amy Llopis Amenta, Sarav Narayanan, Mugdha Deshpande, Rizwana Islam, Shipeng Yuan, Paul McQuade, Christopher T. Winkelmann, and Talakad G. Lohith

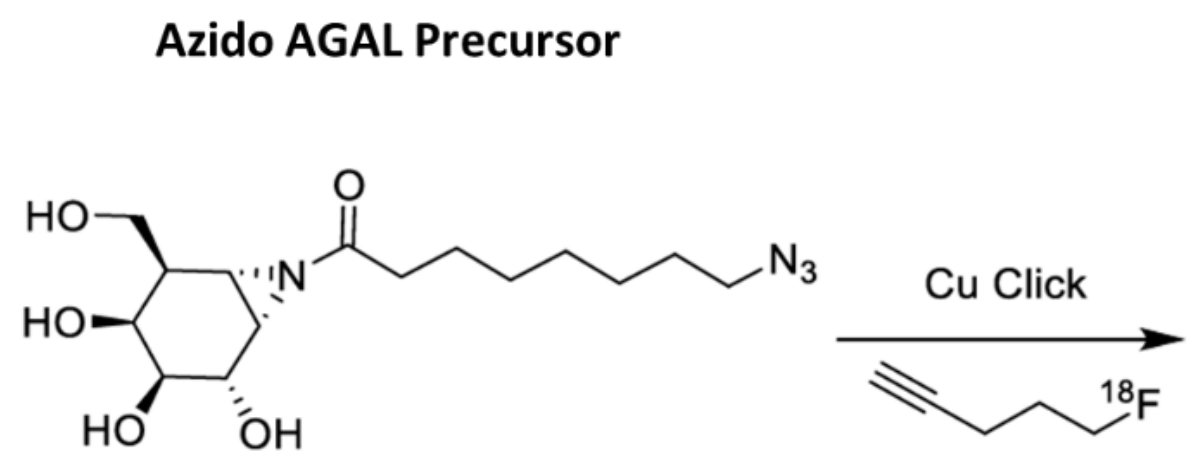

Figure S1: Radiolabeling of the azide-containing AGAL precursor with Fluorine-18, to yield the PET tracer [18F]AGAL after Cu(I)-catalyzed azide–alkyne 1,3-dipolar cycloaddition (CuAAC; click chemistry).

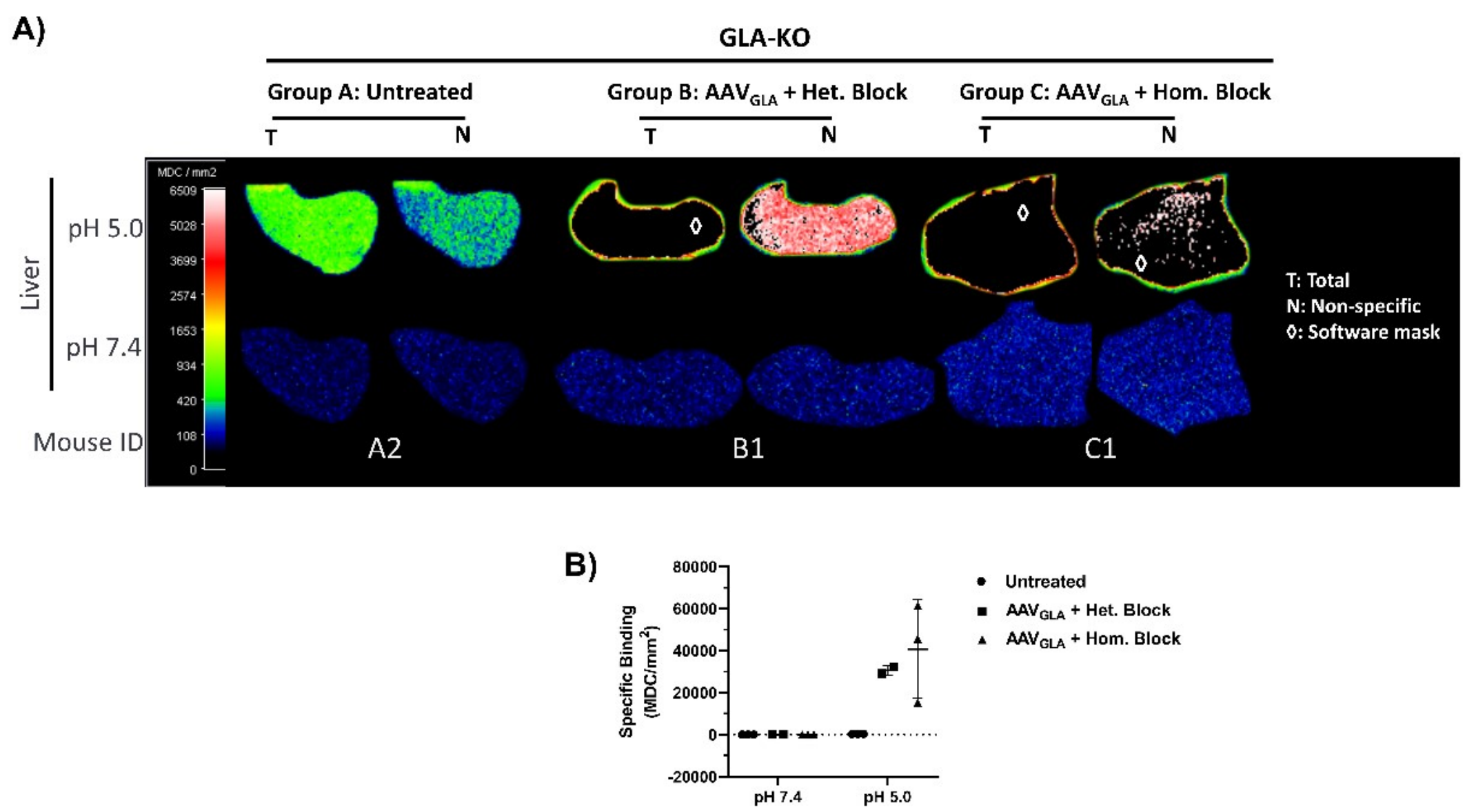

Figure S2. ***In vitro*** **characterization of the [18F]AGAL probe demonstrating binding to human alpha galactosidase A in the liver of transduced Fabry (GLA KO) mice. A:** Representative autoradiography images of mouse liver tissue sections incubated with [18F]AGAL at the corresponding pH, indicating that the probe binds to the enzyme in acidic conditions (pH 5.0). **B:** Quantification of the autoradiography analysis, demonstrating increased specific binding of [18F]AGAL to the livers of Fabry mice transduced with rAAV-ubp-α-GalA. Data are displayed as means and standard deviations with illustration of individual datapoints. (MDC/mm^2: molecular dynamic counts per square millimeter; GLA-KO: GLA knockout Fabry mice; rAAV-ubp-α-GalA: AAV encoding human alpha galactosidase A; Het. and Hom. Block: heterologous and homologous blocking respectively; Software-masked area represents part of the slice where the counts reached saturation).

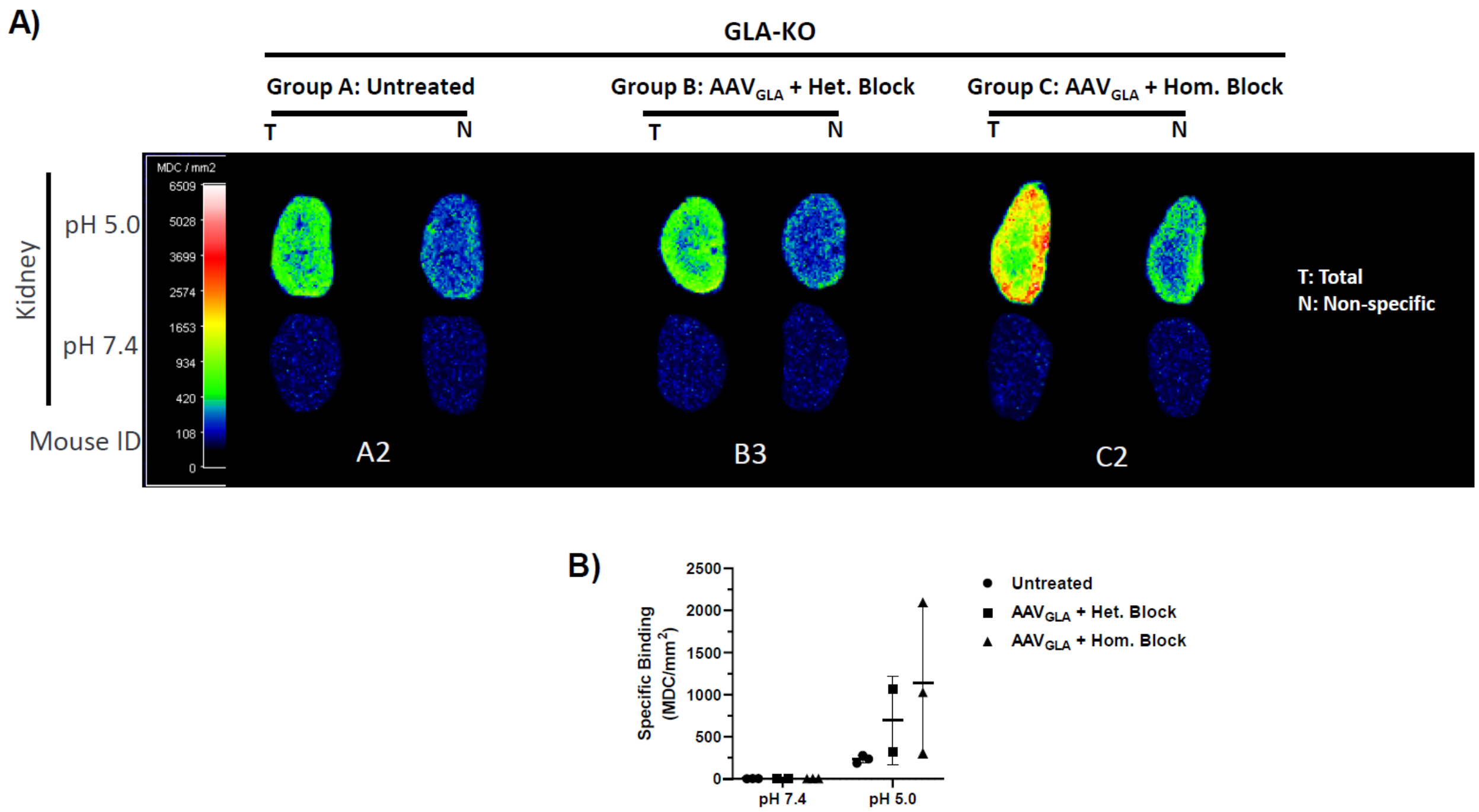

Figure S3. ***In vitro*** **characterization of the [^{18}F]AGAL probe demonstrating binding to human alpha galactosidase A in the kidney of transduced Fabry (GLA KO) mice. A:** Representative autoradiography images of mouse kidney tissue sections incubated with [^{18}F]AGAL at the corresponding pH, indicating that the probe binds to the enzyme in acidic conditions (pH 5.0). **B:** Quantification of the autoradiography analysis, demonstrating increased specific binding of [^{18}F]AGAL to the kidneys of Fabry mice transduced with rAAV-ubp-α-GalA. Data are displayed as means and standard deviations with illustration of individual datapoints. (MDC/mm^2: molecular dynamic counts per square millimeter; GLA-KO: GLA knockout Fabry mice; rAAV-ubp-α-GalA: AAV encoding human alpha galactosidase A; Het. and Hom. Block: heterologous and homologous blocking respectively).

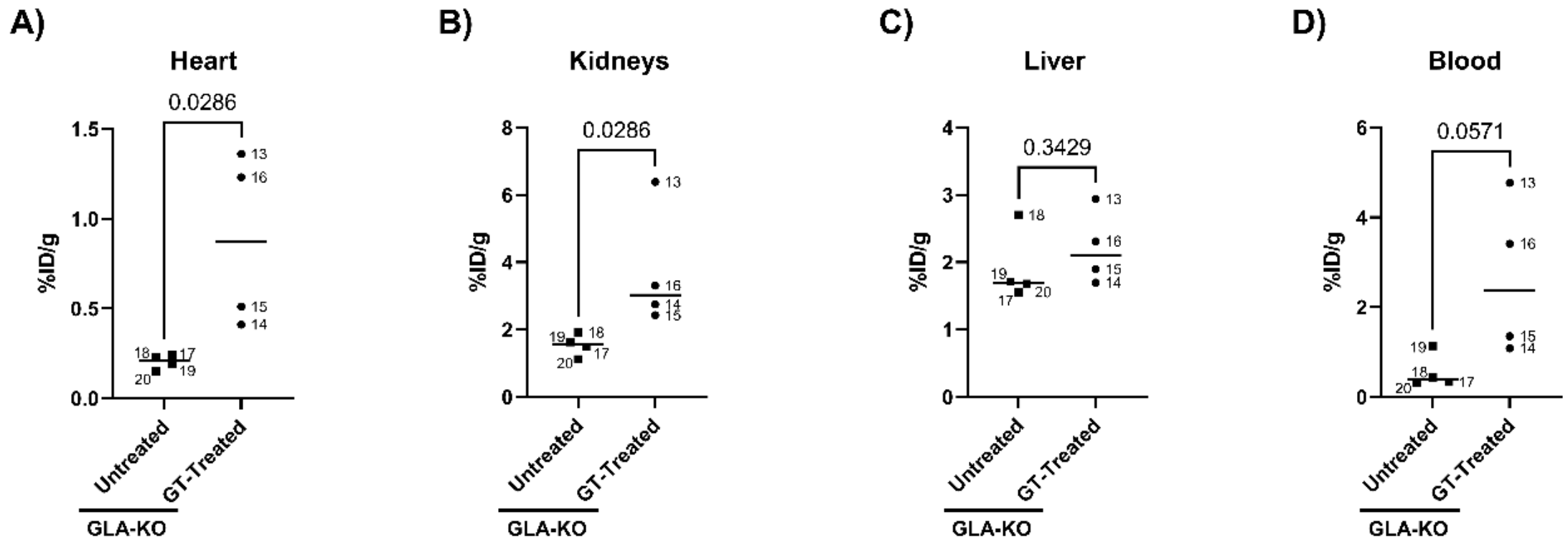

Figure S4. ***Ex vivo*** **evaluation of the uptake of the [^{18}F]AGAL probe in Fabry (GLA KO) mice.** Quantification of the tracer's levels in the **(A)** heart, **(B)** kidneys, **(C)** liver, and **(D)** blood using a gamma counter. Data are displayed as means with illustration of individual datapoints. The animals' identifiers are next to the corresponding datapoint. Statistical significance was determined using the Mann-Whitney test, and the p value is stated on top of the horizonal bar that compares the two groups. (%ID/g: percent injected dose of [^{18}F]AGAL per gram of tissue/blood; GLA-KO: GLA knockout Fabry mice; GT-Treated: animals transduced with AAV encoding human alpha galactosidase A).

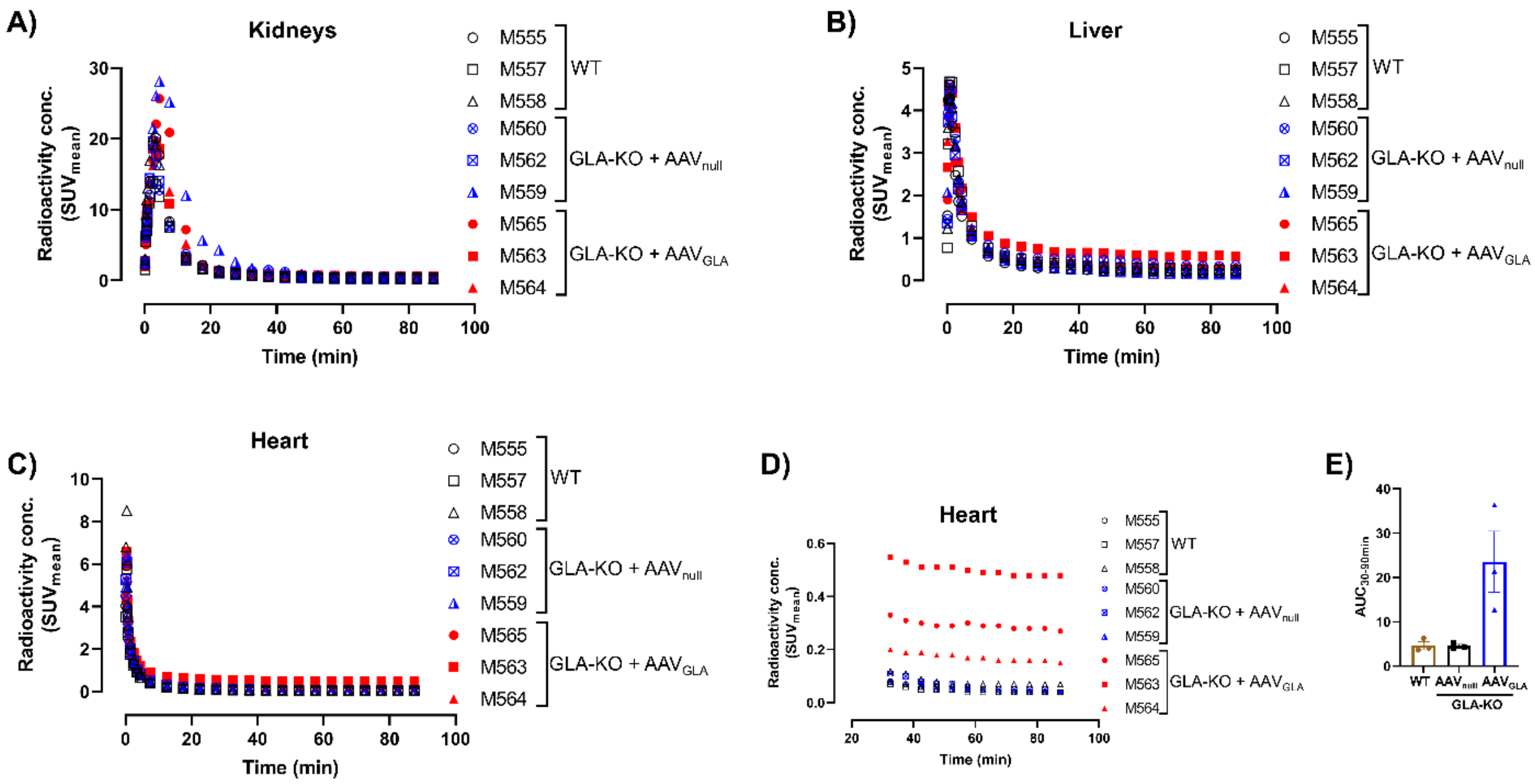

Figure S5. Time-activity uptake curves of major organs in GLA-KO mice 6 weeks after receiving gene therapy. A-C: PET-based determination of the kinetics of $[^{18}F]$AGAL in kidneys, liver, and heart. The animals' identifiers are depicted in the plots. **D:** $[^{18}F]$AGAL uptake in the heart from 30to 90 minutes, showing higher retention in the AAV_{GLA}-treated mice. **E:** Fabry mice that underwent gene therapy that encodes human alpha galactosidase A have higher $[^{18}F]$AGAL uptake in their hearts. Data are displayed as means and standard deviations with illustration of individual datapoints. (SUV_{mean}: mean standardized uptake value; WT: wildtype; GLA-KO: GLA knockout Fabry mice; AAV_{GLA}: AAV encoding human alpha galactosidase A; AAV_{null}: AAV not encoding a therapeutic protein; AUC: area-under-curve).

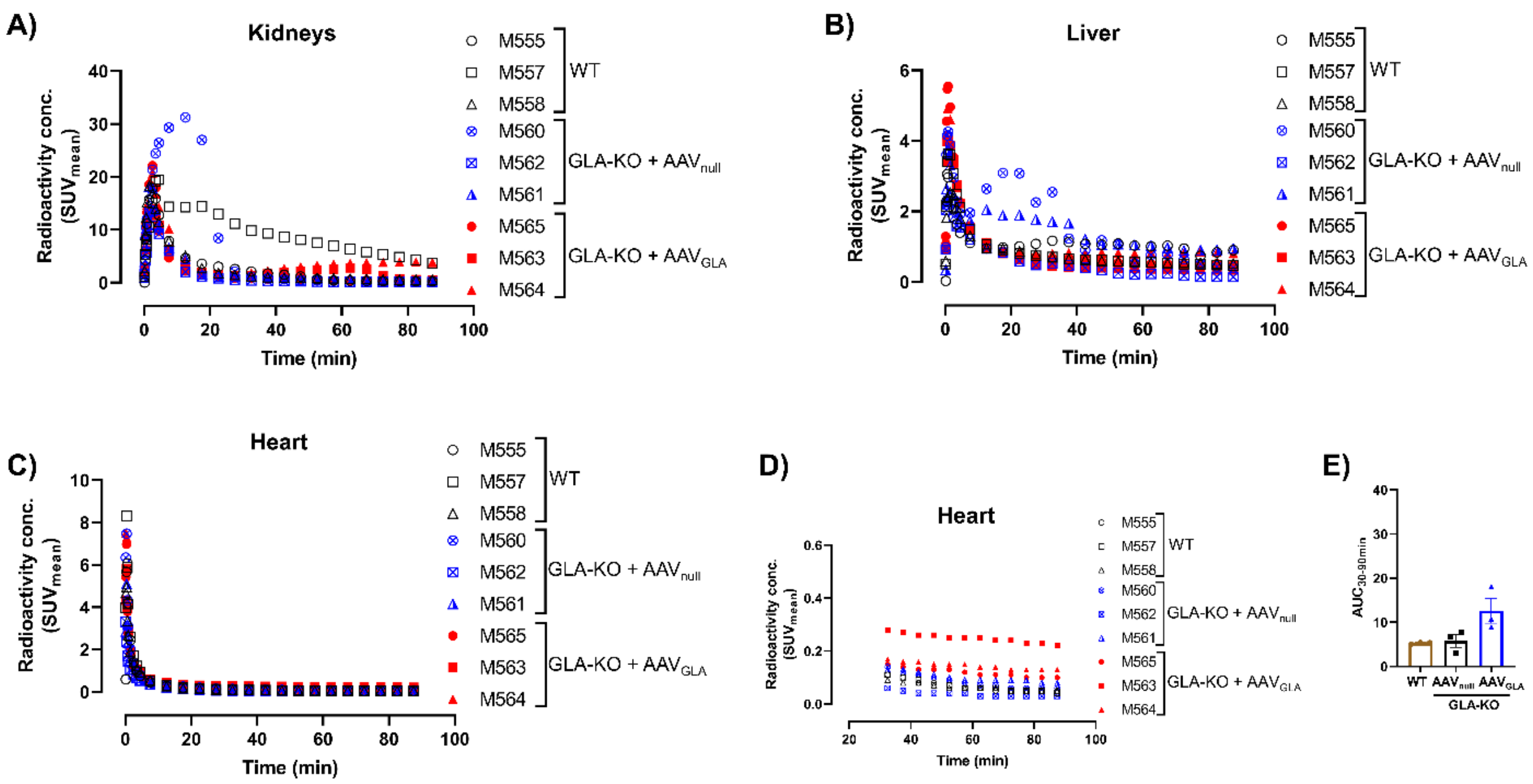

Figure S6. Time-activity uptake curves of major in GLA-KO mice 26 weeks after receiving gene therapy. A-C: PET-based determination of the kinetics of [^{18}F]AGAL in kidneys, liver, and heart. The animals' identifiers are depicted in the plots. **D:** [^{18}F]AGAL uptake in the heart from 30 to 90 minutes, showing higher retention in the AAV_{GLA}-treated mice. **E:** Fabry mice that underwent gene therapy that encodes human alpha galactosidase A have higher [^{18}F]AGAL uptake in their hearts. Data are displayed as means and standard deviations with illustration of individual datapoints. (SUV_{mean}: mean standardized uptake value; WT: wildtype; GLA-KO: GLA knockout Fabry mice; AAV_{GLA}: AAV encoding human alpha galactosidase A; AAV_{null}: AAV not encoding a therapeutic protein; AUC: area-under-curve).

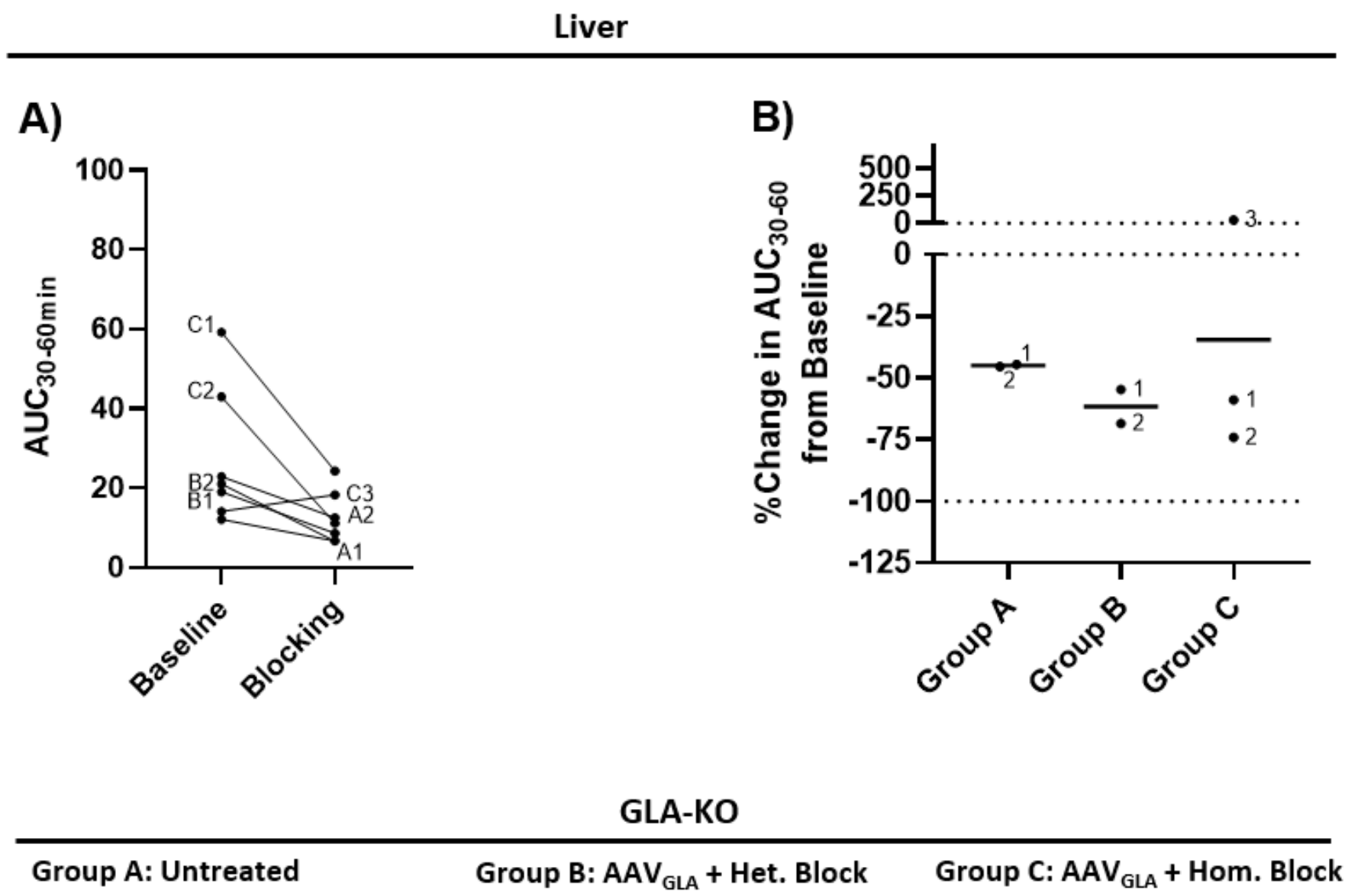

Figure S7. ***In vivo*** **determination of the specificity of [^{18}F]AGAL binding in the liver of gene-therapy-treated Fabry (GLA-KO) mice. A:** Quantification of the PET signal in the liver, and **(B)** determination of the changes in the tracer's uptake due to the blocking agent. Each datapoint depicts the corresponding animal identifiers. Each group's horizontal bar in panel **(B)** depicts the group mean. Heterologous block was done with Migalastat, and homologous block was achieved with [^{19}F]AGAL (Migalastat and [^{19}F]AGAL were dosed at 1 mg/kg). (GLA KO: GLA knockout Fabry mice; AUC_{30-60}: area-under-curve of 30 to 60 minutes post tracer dosing; AAV_{GLA}: AAV encoding human alpha galactosidase A; Het. and Hom. Block: heterologous and homologous blocking respectively).

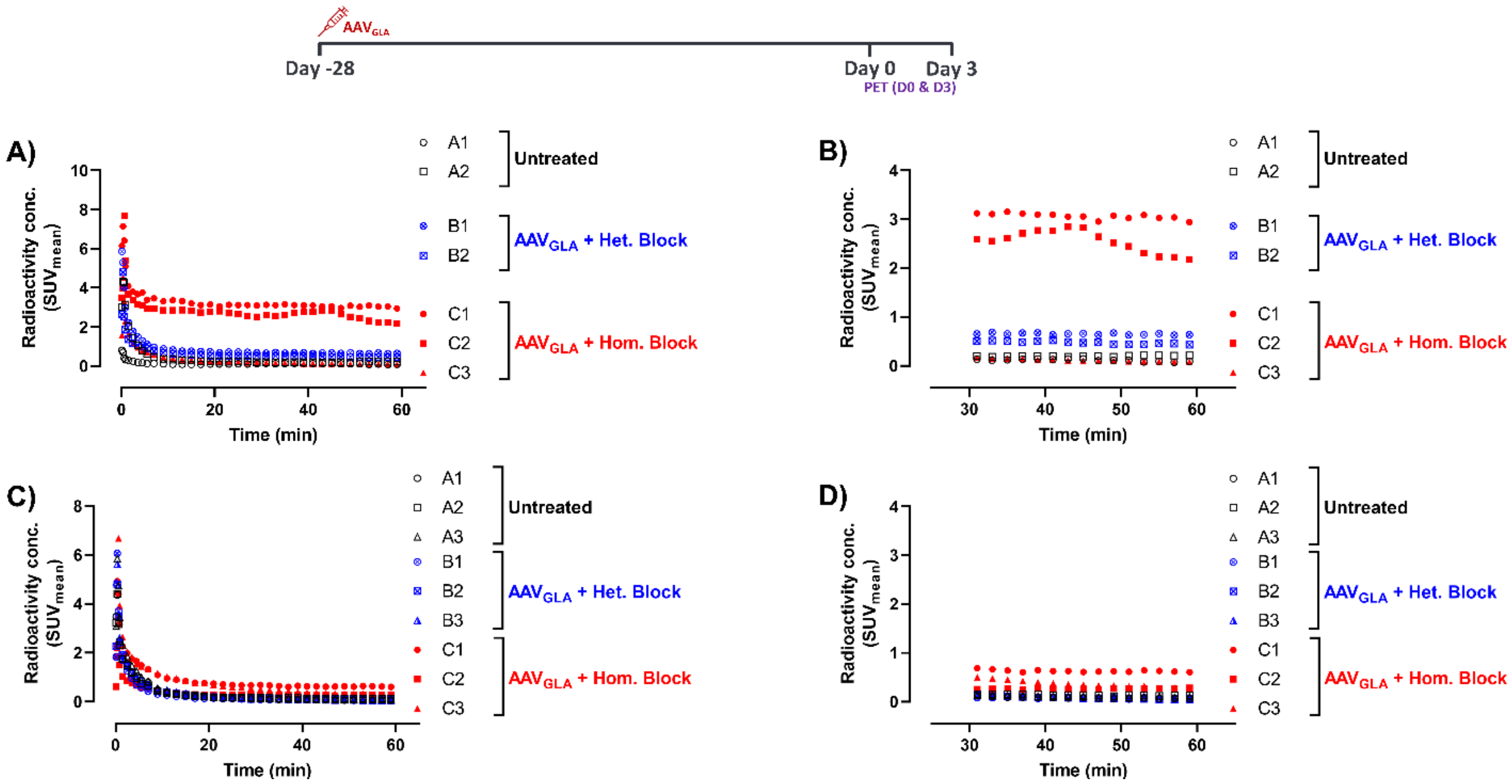

Figure S8. Heart time-activity curves in GLA-KO mice after receiving gene therapy, where [18F]AGAL was administered after the blocking agent. PET-based determination of the kinetics of [18F]AGAL in the heart **(A-B)** on Imaging Day 0 (28 days after dosing of AAV_{GLA} as baseline) and **(C-D)** on Imaging Day 3 (following administration of a heterologous or homologous blocking agent. Heterologous block was done with Migalastat, and homologous block was achieved with [19F]AGAL (Migalastat and [19F]AGAL were dosed at 1 mg/kg). Reduction in the uptake of the tracer due to blocking is observed on Imaging Day 3. The animals' identifiers are depicted in the plots. (SUV_{mean}: mean standardized uptake value; GLA-KO: GLA knockout Fabry mice; AAV_{GLA}: AAV encoding human alpha galactosidase A; Het. and Hom. Block: heterologous and homologous blocking respectively).

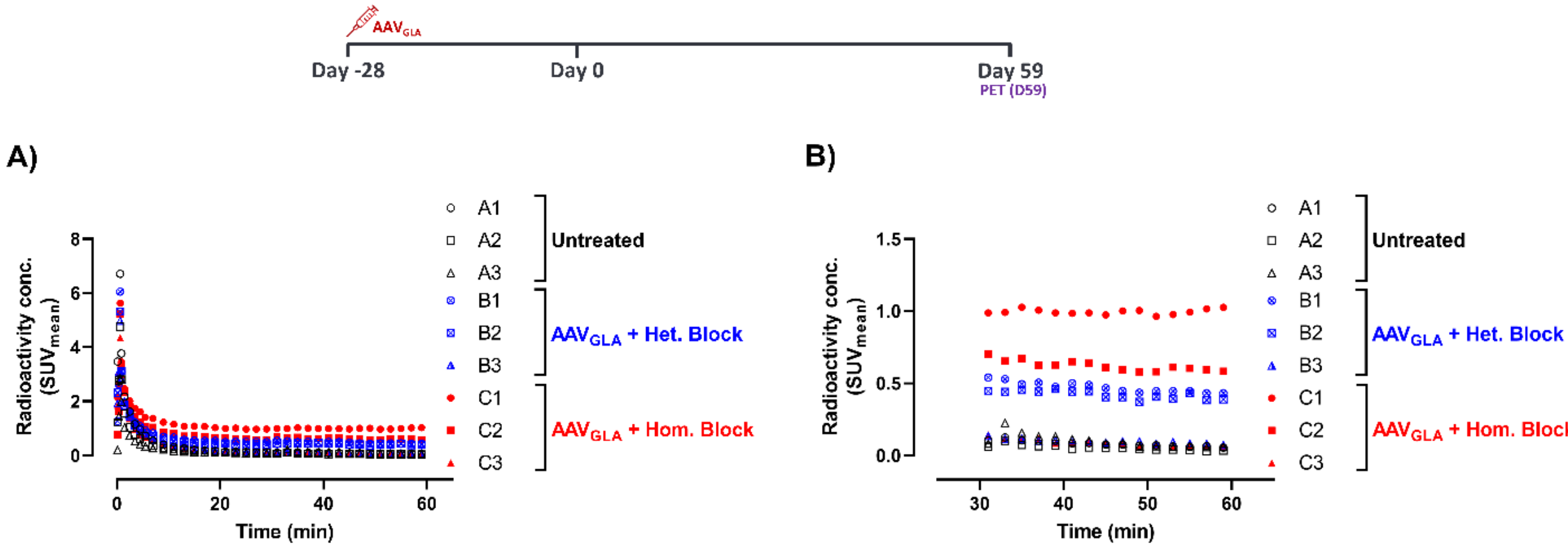

Figure S9. Heart time-activity curves in GLA-KO mice after receiving gene therapy, where [^{18}F]AGAL was administered 30 minutes before the blocking agent. PET-based determination of the kinetics of [^{18}F]AGAL in the heart **(A-B)** on Imaging Day 59 (59 days after dosing of AAV_{GLA} as baseline). Heterologous block was done with Migalastat, and homologous block was achieved with [^{19}F]AGAL (Migalastat and [^{19}F]AGAL were dosed at 1 mg/kg). AAV_{GLA}-treated mice maintained high tracer uptake, suggesting that the tracer cannot be displaced by the blocking compound. The animals' identifiers are depicted in the plots. (SUV_{mean}: mean standardized uptake value; GLA-KO: GLA knockout Fabry mice; AAV_{GLA}: AAV encoding human alpha galactosidase A; Het. and Hom. Block: heterologous and homologous blocking respectively).

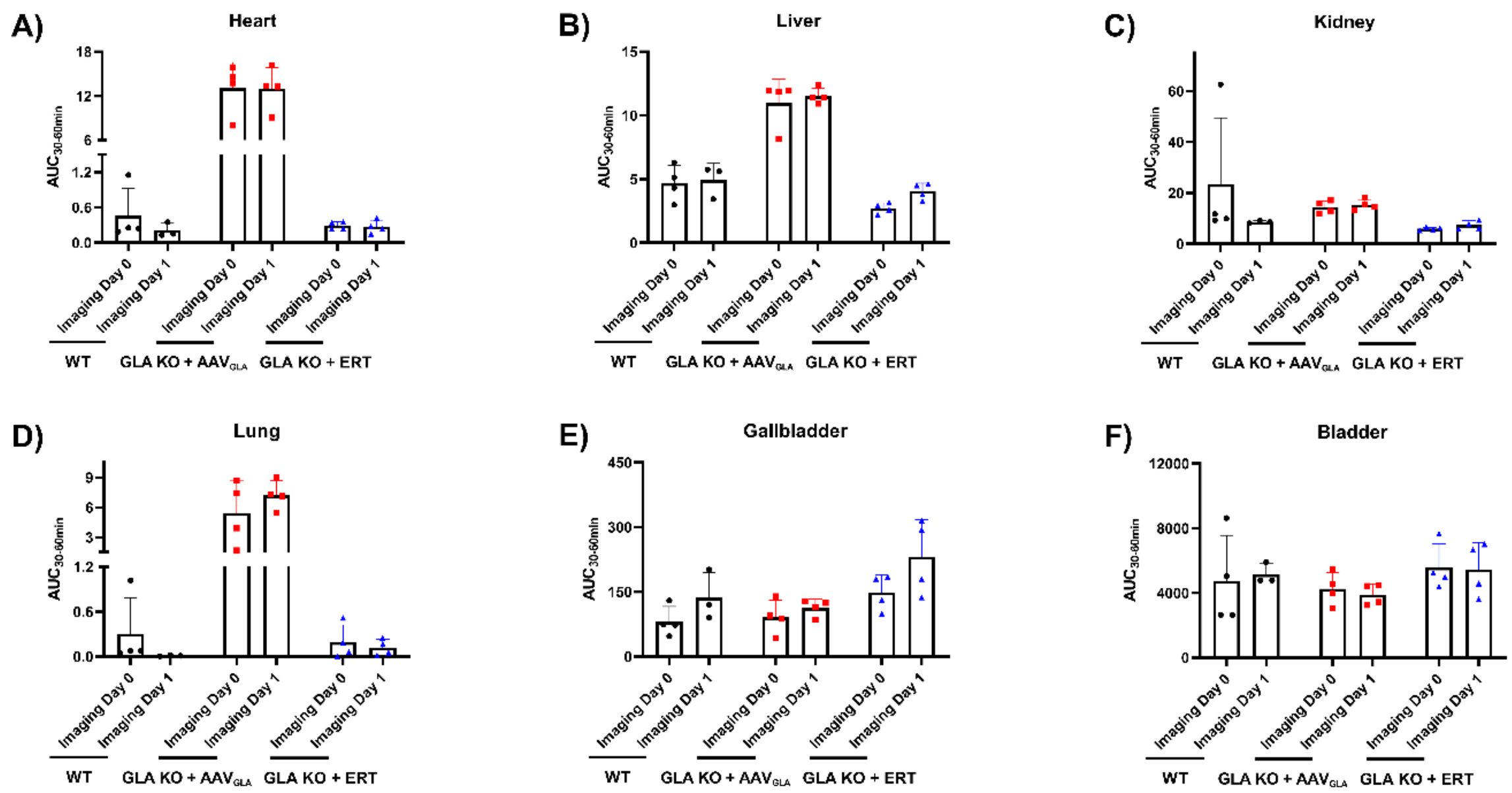

Figure S10. ***In vivo*** **comparison of [^{18}F]AGAL uptake in gene-therapy-** ***vs*** **enzyme-replacement-treated Fabry (GLA KO) mice.** PET-based determination of the retention of [^{18}F]AGAL in **(A)** the heart, **(B)** liver, **(C)** kidney, **(D)** lung, **(E)** gallbladder, and **(F)** bladder. Higher uptake is observed in the heart, liver, and lung of gene-therapy-treated animals. Data are displayed as means and standard deviations. (AUC_{30-60}: area-under-curve of 30 to 60 minutes post tracer dosing; WT: wildtype mice; GLA-KO: GLA knockout Fabry mice; AAV_{GLA}: AAV encoding human alpha galactosidase A; ERT: enzyme replacement therapy).

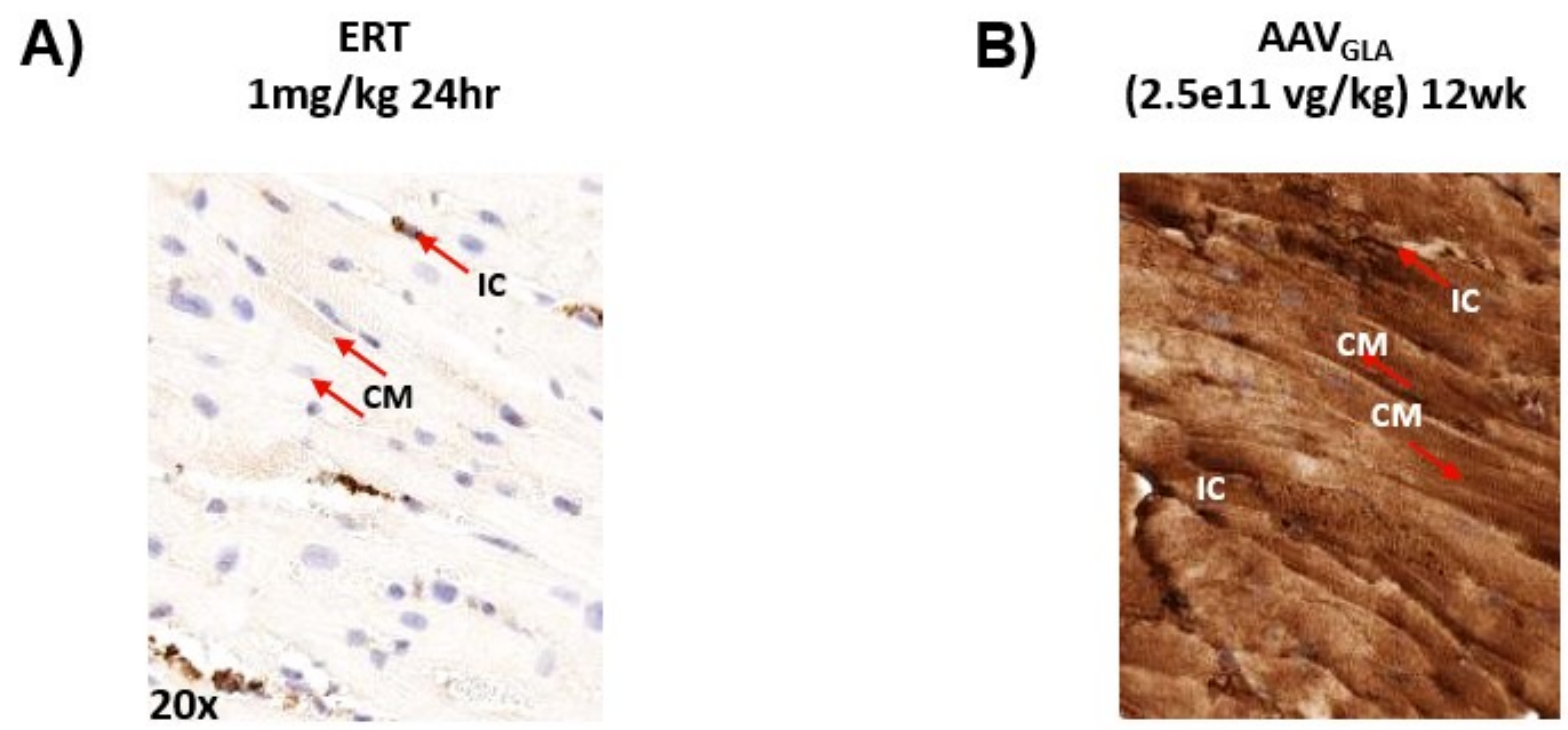

Figure S11. Immunohistochemical evaluation of the heart of Fabry (GLA KO) mice for human alpha galactosidase A. Representative immunohistochemistry images of heart tissue sections of animals that didn't undergo PET imaging are shown. **A:** alpha galactosidase A is detected 24 hours after enzyme replacement therapy (ERT). **B:** The enzyme is detected in multiple cells 12 weeks after gene therapy, with pronounced staining observed in the mice that received AAV_{GLA}. (AAV_{GLA}: AAV encoding human alpha galactosidase A; CM: cardiomyocytes; IC: interstitial cells; α-Gal A positive staining (brown)).

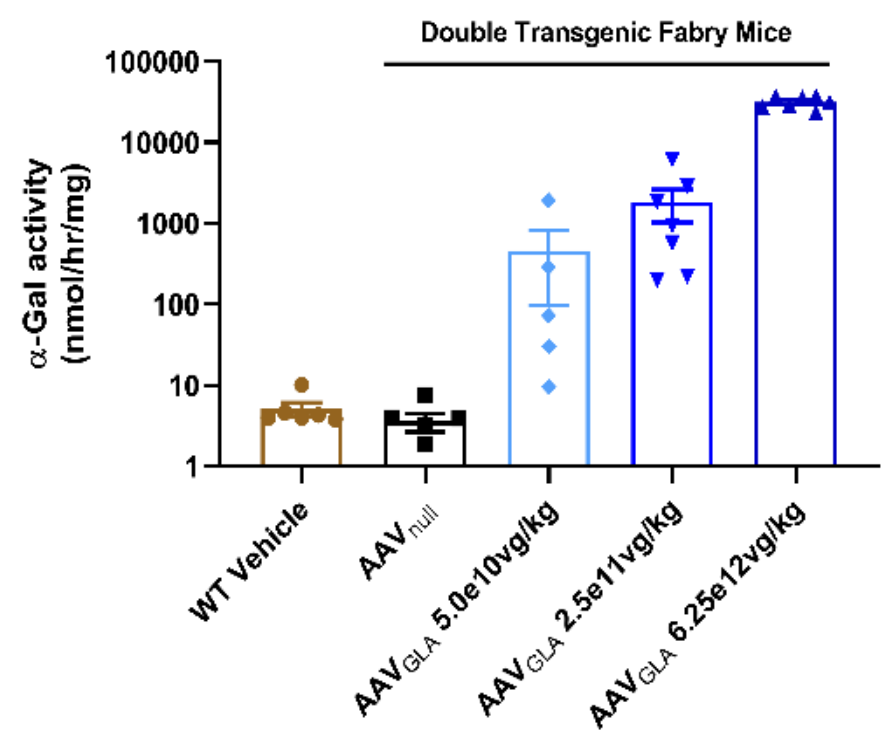

Figure S12. GLA activity in the heart after transduction with different AAV_{GLA} titers. Gene therapy of double transgenic Fabry mice (G3Stg/GLA KO) induces dose-dependent expression of active alpha galactosidase A in the heart. Data are displayed as means and standard deviations with illustration of individual datapoints on panel. (WT: wildtype mice; AAV_{GLA}: AAV encoding human alpha galactosidase A; AAV_{null}: AAV not encoding a therapeutic protein; vg/kg: viral genome copies per kilogram).

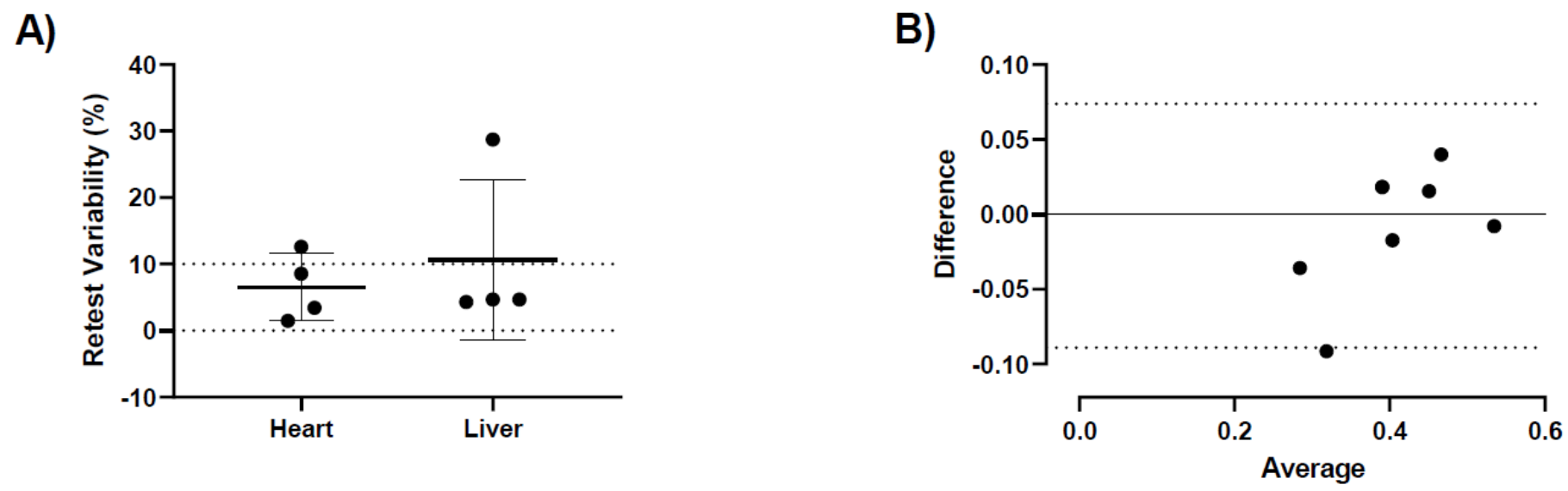

Figure S13. Retest variability and Bland-Altman plot of [^{18}F]AGAL. A: The tracer's retest variability in the heart and liver was determined using AAV_{GLA}-treated Fabry mice that were imaged on two consecutive days (Imaging Day 0 and 1). Tracers with retest variability under 10 % are considered excellent. **B:** In the Bland-Altman plot the solid middle line denotes the bias from zero, whereas the dashed lines depict the 95 % limits of agreement (i.e. bias ± 1.96 × standard deviation). Data are displayed as means and standard deviations with illustration of individual datapoints on panel A. (SUV: standardized uptake value; AAV_{GLA}: AAV encoding human alpha galactosidase A).

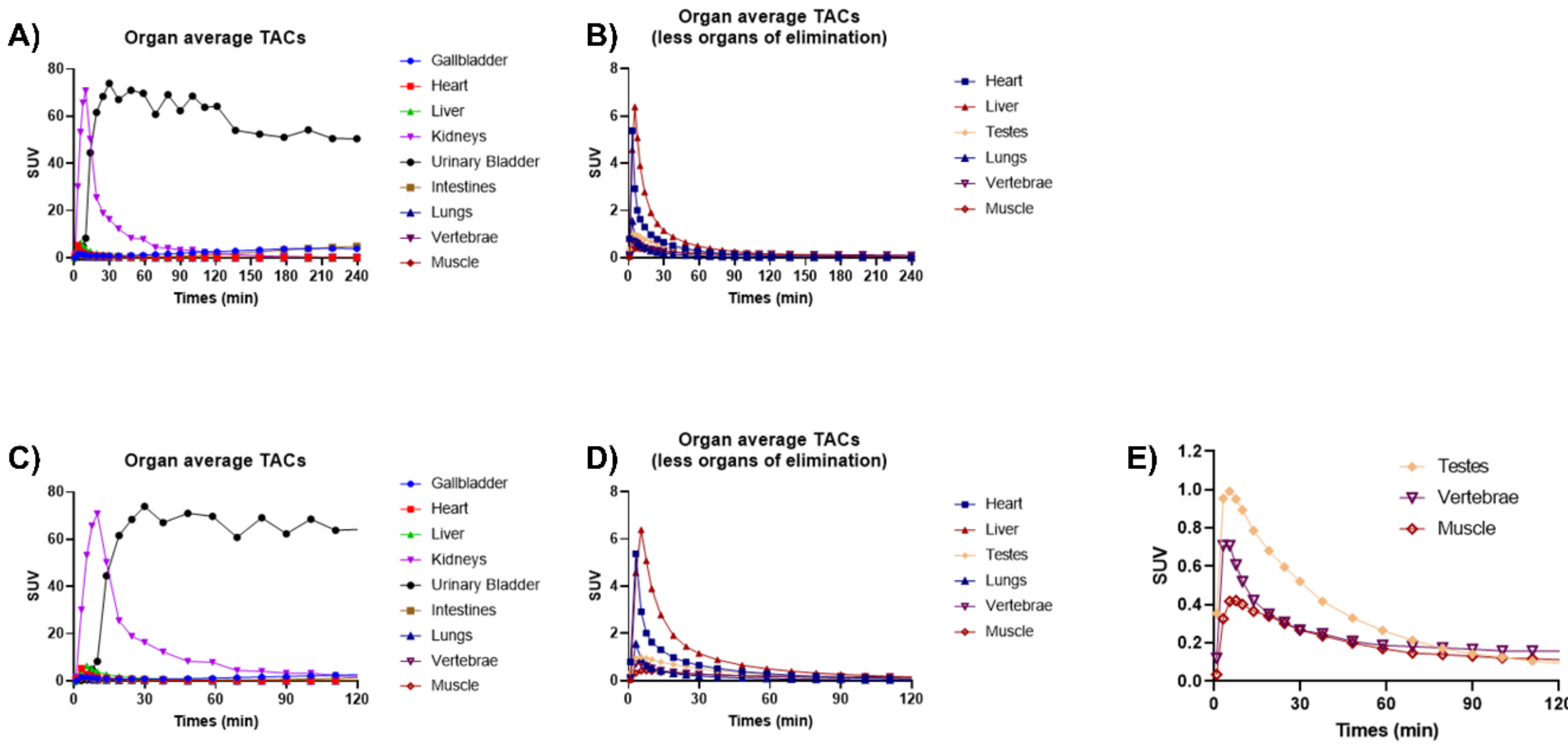

Figure S14. Time-activity uptake curves of [^{18}F]AGAL in male rhesus monkey (*Macaca mulatta*). PET-based determination of the kinetics of [^{18}F]AGAL in select organs following administration of the tracer. Panels **(A-B)** depict the kinetics for the duration of the scan, whereas panes **(C-E)** show [^{18}F]AGAL's kinetics up to 2 hours after intravenous administration. (SUV: standardized uptake value; TACs: time-activity curves).
