# Supplementary material for: Longitudinal imaging of therapeutic enzyme expression after gene therapy for Fabry disease using positron emission tomography and the radiotracer [18F]AGAL
Source: Mol Ther. 2024 Nov 19;33(9):4381–93. doi: 10.1016/j.ymthe.2024.11.021 (PMC12432858; doi:10.1016/j.ymthe.2024.11.021)
Supplement: Supplementary file 2 [file mmc2.pdf]

Molecular Therapy
Original Article

Longitudinal imaging of therapeutic enzyme expression after gene therapy for Fabry disease using positron emission tomography and the radiotracer [^{18}F]AGAL

Charalambos Kaittanis,[1] Tyler Teceno,[1] Ashley Knight,[1] Yoann Petibon,[1] Phil Sandoval,[1] Lawrence Cohen,[1] Shin Hye Ahn,[2] Anthony P. Belanger,[2] Louise M. Clark,[3] Quang-De Nguyen,[3] Wanida Ruangsiriluk,[1] Shreya Mukherji,[1] Cristian C. Constantinescu,[4] Amy Llopis Amenta,[4] Sarav Narayanan,[1] Mugdha Deshpande,[1] Rizwana Islam,[1] Shipeng Yuan,[1] Paul McQuade,[1] Christopher T. Winkelmann,[1] and Talakad G. Lohith[1]

[1]Takeda Pharmaceutical Company Limited, Cambridge, MA 02142, USA; [2]Molecular Cancer Imaging Facility, Dana-Farber Cancer Institute, Boston, MA 02215, USA; [3]Lurie Family Imaging Center, Dana-Farber Cancer Institute, Boston, MA 02215, USA; [4]Invicro, New Haven, CT 06510, USA

Longitudinal, non-invasive, *in vivo* monitoring of therapeutic gene expression is an unmet need for gene therapy (GT). Positron emission tomography (PET) radiotracers designed to bind to therapeutic proteins may provide a sensitive imaging platform to guide treatment response and dose optimization in GT. Herein, we evaluate a novel PET tracer ([^{18}F]AGAL) for targeting α-galactosidase A (GLA), an enzyme deficient in Fabry disease. *Gla* knockout mice were subjected to either GT with an adeno-associated virus encoding the human GLA (AAV_{GLA}) or recombinant GLA for enzyme replacement studies. PET imaging, *ex vivo* autoradiography, biochemical analyses and radiation dosimetry were performed. [^{18}F]AGAL exhibited pH-dependent binding to GLA, suggesting recognition of the active enzyme residing within the acidified lysosomes. Imaging studies in the Fabry mouse model showed quick renal clearance with high radioactive uptake in the heart at 6 weeks that was sustained for 26 weeks after a single administration of AAV_{GLA}, indicating effective and durable transgene expression from GT. Good concordance was achieved between *in vivo* PET imaging and *ex vivo* quantification of GLA levels in biofluids and tissues. Biodistribution and dosimetry in non-human primate showed acceptable radiation exposure for multiple injections, demonstrating its potential for translation to clinical trial use.

INTRODUCTION

Fabry disease is an X-linked genetic disorder that occurs due to the mutations in the *GLA* gene, which encodes the lysosomal enzyme α-galactosidase A (GLA). Because GLA performs the rate-limiting step in the degradation of glycosphingolipids, mutations that render the enzyme inactive cause accumulation of its substrate globotriaosylceramide (Gb_3) in the lysosomes of various organs. The disease phenotype includes but is not limited to peripheral neuropathy, gastrointestinal (GI) pain, enteric dysmotility, renal failure, stroke, and cardiac hypertrophy with or without fibrosis. Among the clinical presentations of Fabry disease, the development and advancement of cardiovascular pathology is the leading cause of mortality, accounting for 75% of all Fabry deaths.[1] This is primarily attributed to limitations of the current standard treatments for Fabry disease, which do not effectively ameliorate the lysosomal burden in the heart. Enzyme replacement therapy (ERT), including agalsidase alpha (Replagal, Takeda) and agalsidase beta (Fabrazyme, Sanofi), slow disease progression in some organs but not the heart, plausibly due to poor tissue penetration of ERT and immunogenicity affecting the stability of ERT's recombinant protein in the blood.[2] Other disease-modifying therapies, such as the oral chaperone migalastat (Galafold, Amicus Therapeutics), can be used in patients with certain mutations to partly restore the activity of GLA, although more data are needed to determine the drug's long-term efficacy in the heart. Furthermore, native agalsidase alpha and beta's modest lysosomal half-life limit their therapeutic efficacy,[3] hence the development of next-generation Fabry therapies that reintroduce the functional enzyme in the heart is critical.

Adeno-associated virus (AAV) vectors are among the most promising vehicles for effective gene delivery, because of their ability to transduce different organs, long-term sustained expression of the therapeutic protein, low immunogenicity, and good tolerance, with the consensus being that AAVs do not cause any human diseases.[4] The ability to generate customized therapeutics by engineering the vector genome and capsids, combined with its safety profile has made AAV-based gene therapy a modality of choice for several

Received 15 April 2024; accepted 15 November 2024;
https://doi.org/10.1016/j.ymthe.2024.11.021.

Correspondence: Talakad G. Lohith, Takeda Pharmaceutical Company Limited, Cambridge, MA 02142, USA.
E-mail: talakad.lohith@takeda.com

Published by Elsevier Inc. on behalf of The American Society of Gene and Cell Therapy.
This is an open access article under the CC BY license (http://creativecommons.org/licenses/by/4.0/).

therapeutic interventions. This approach led to the first EMA approval of an AAV-based therapy in 2012 followed by the first FDA approval in 2017, and the dawn of gene therapy for monogenic diseases.[5]

The success of gene therapy relies on the durable expression and activity of the transgene in target tissues. Current methods measure transgene expression and activity either in body fluids, which may or may not reflect target organ levels, or from biopsies, which are invasive and prone to sampling errors. Positron emission tomography (PET) is a non-invasive, quantitative, *in vivo* imaging method with high sensitivity, and provides accurate estimates of the concentration of a radiolabeled probe that targets with high biologic specificity a protein of interest in tissues. Although elegant molecular imaging efforts using PET have focused on the use of reporter genes to indirectly report the transgene expression after gene therapy,[6,7] it is more desirable to develop PET tracers that can directly interrogate the levels and activity of a therapeutic protein longitudinally at whole-body level and in particular to the organs of interest that are affected and less amenable for biopsies.

We hypothesized that a small-molecule PET radiotracer that specifically binds to GLA could interrogate the levels and activity of GLA *in vivo* in terms of location, magnitude, and durability following systemic administration of the AAV vector and the resultant expression of GLA in the liver, heart, and other tissues in the body. [^{18}F]AGAL is a novel PET tracer that has high nanomolar affinity and high selectivity to GLA and has shown rapid clearance and suitable kinetics *in vivo* in wild-type mice.[8] Here, we used AAV_{GLA}, a gene therapy vector that consists of an AAV9 capsid and a genome that contains the chicken β-actin promoter that drives expression of human GLA in various tissues in the body, including the liver and heart. We aimed to demonstrate that [^{18}F]AGAL could detect AAV9-mediated GLA expression in the heart longitudinally and characterized the tracer's uptake and kinetics in a mouse model of Fabry disease. In addition, we characterized the tracer biodistribution and radiation dosimetry in a non-human primate (NHP) to facilitate the translation of [^{18}F]AGAL for use as a pharmacokinetic/pharmacodynamic biomarker in clinical trials of GLA-targeted gene therapies.

RESULTS

In vitro evaluation of the [^{18}F]AGAL probe

We recently developed a fluorine-18 radiotracer ligand of GLA ([^{18}F]AGAL: fluorine-18 for α-galactosidase A ligand), by conjugating an azido aziridine precursor to ^{18}F-labeled pentyne using copper-catalyzed click chemistry (Figure S1).[8] Stability analysis showed that the rate of degradation of a radio-concentration of ~32 mCi/mL [^{18}F]AGAL was ~7% over a 6-h period following the end of synthesis, and ~5% degradation if the radio-concentration was ~20 mCi/mL. The radiochemical purity for all production runs used in our studies was greater than 90%, with a typical production of 100 mCi of tracer (molar activity ≥ 1,000 mCi/μmol). We then determined that this probe could detect human GLA in cardiac tissue of AAV_{GLA}-transduced mice (Figure 1). Minimal binding of [^{18}F]AGAL was observed in the heart tissues of wild-type mice, and Fabry (GLA KO) mice that were dosed with the control null vector not encoding a therapeutic protein (Figure 1A). Quantification of the autoradiography data identified that the probe had 262-fold higher specific binding in heart tissues from the AAV_{GLA}-treated mice than the corresponding controls (Figure 1B). Interestingly, the heart tissue of wild-type mice exhibited slightly elevated specific binding than that of AAV_{null}-treated Fabry mice, which may be attributed to the modest lysosomal half-life of native GLA as mentioned above. We further determined that this radiotracer bound to GLA at pH 5.0 but not at physiological pH (pH 7.4), suggesting that the probe likely binds to the active form of GLA that resides within the lysosomes and may have minimal association with it in circulation (Figure 1A). This observation is in accordance with the mechanism of interaction between fluorescent aziridines and GLA that occurs in acidic conditions, but not in neutral or basic conditions.[9] In addition to the heart, we found that [^{18}F]AGAL could detect its enzyme target in the liver and kidney tissue sections of AAV_{GLA}-transduced Fabry mice and maintain its pH-dependent binding (Figures S2 and S3). Using cardiac tissue sections from AAV_{GLA}-treated animals we determined at two different concentration ranges that the probe has a dissociation constant K_D of ~20 nM for its target at pH 5.0 (Figures 1C and 1D). This is comparable with the dissociation constant of a fluorescent aziridine for GLA determined in the absence of probe-target pre-incubation,[9] and showed that our radiolabeling strategy did not affect the probe's affinity to its target.

PET and [^{18}F]AGAL monitor gene therapy in a mouse model of Fabry disease

We next investigated [^{18}F]AGAL's ability to monitor the expression of GLA *in vivo*, using a wild-type and mouse model of Fabry disease. Maximum intensity projection (MIP) PET images, where the maximum signal at a given voxel over a time interval is depicted, showed distinct differences between AAV_{GLA}-treated and control mice (Figures 2A and 2B). PET imaging at 6 weeks after AAV transduction revealed significant uptake of the tracer in the heart of the AAV_{GLA}-treated mice, with nominal signal in the heart of wild-type and control null-vector-treated Fabry animals (Figure 2A). The gene-therapy-treated animals had higher signal than their control counterparts at 26 weeks after administration of the AAV (Figure 2B), demonstrating [^{18}F]AGAL's ability to longitudinally detect the transgene's levels *in vivo* and AAV's potential to robustly express a therapeutic protein in a major organ of interest after a single administration of the gene therapy. We confirmed the PET data through *ex vivo* analyses (Figures 2C–2F), and determined that there was a positive correlation between [^{18}F]AGAL's uptake in the heart and both GLA activity in the heart and serum, as well as between liver or kidney and serum. This longitudinal evaluation showed that the levels of GLA decreased in the AAV_{GLA}-treated animals from week 6 to week 26 (Figure 2D), and this was reflected in the PET signal measured in the heart (Figure 2C), as well as visually in the MIP images (Figures 2A and 2B). Like the heart, lower GLA expression at week 26 was associated with a decreased PET signal in other organs (Figures 2E and 2F). It should be noted that these analyses were

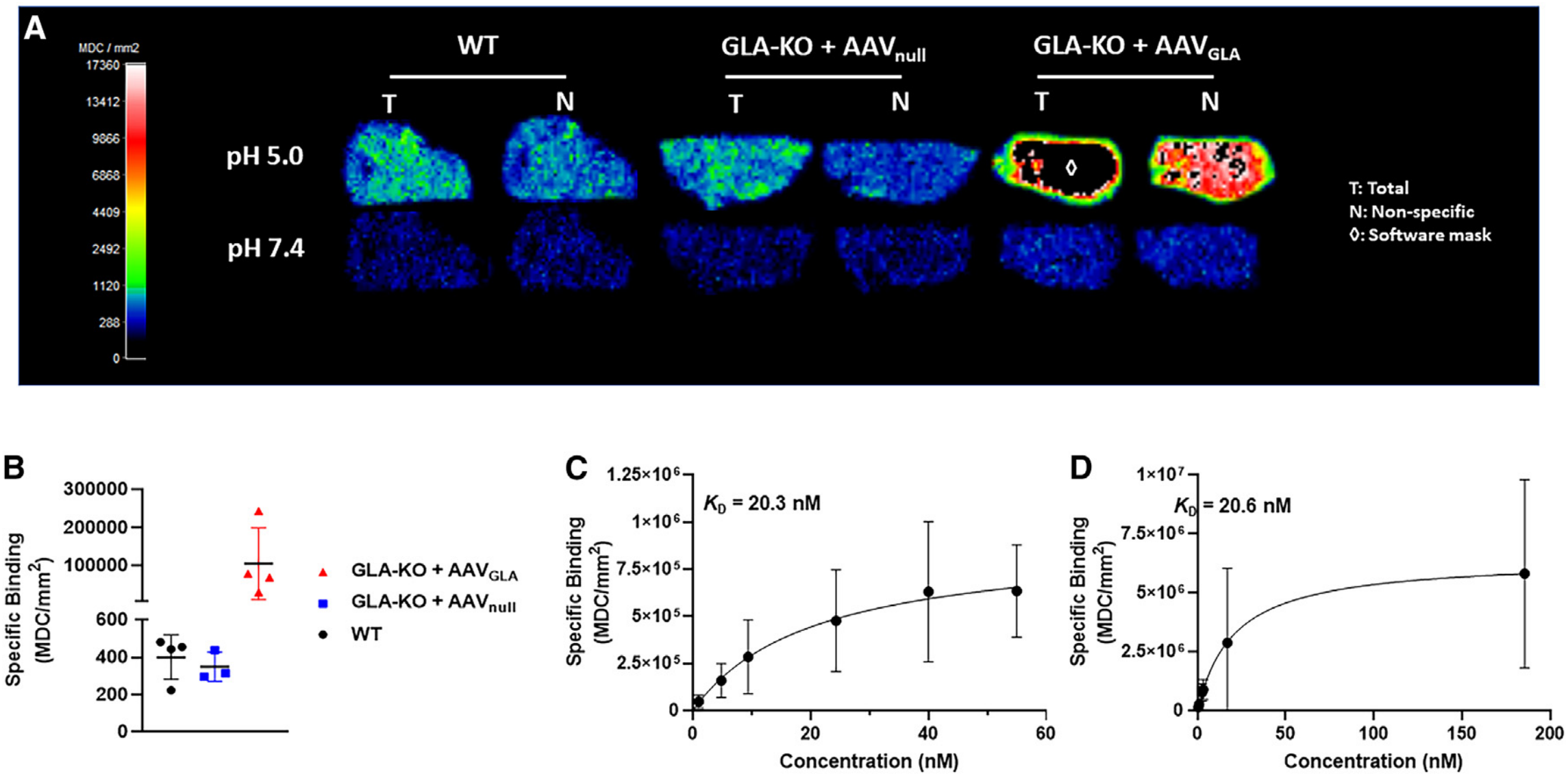

Figure 1. *In vitro* characterization of the [^{18}F]AGAL probe demonstrating binding to human GLA

(A) Representative autoradiography images of murine heart slices incubated with 35 nM of [^{18}F]AGAL at the corresponding pH, indicating that the probe binds to the enzyme in acidic conditions (pH 5.0). (B) Quantification of the autoradiography analysis of (A), demonstrating 262-fold increased specific binding of [^{18}F]AGAL to the hearts of Fabry mice transduced with AAV_{GLA}. (C and D) Determination of the equilibrium dissociation constant (K_D) of [^{18}F]AGAL using heart slices from AAV_{GLA}-transduced Fabry mice and different concentrations of [^{18}F]AGAL in two independent studies. Data are displayed as means and standard deviations with illustration of individual datapoints on (B). MDC/mm^2, molecular dynamic counts per square millimeter; WT, wild-type; GLA-KO, GLA knockout Fabry mice; AAV_{GLA}, AAV encoding human α-galactosidase A; AAV_{null}, AAV not encoding a therapeutic protein. Software-masked area represents part of the slice where the counts reached saturation.

performed at the end of the study at 26 weeks post-AAV injection, thus potential decreases in the levels of GLA between weeks 6 and 26 may be attributed to distinct events that affect transgene expression, such as promoter hypermethylation, that may contribute to intracohort variability.[10] Considering that cardiomyocytes have very low turnover, it is less likely that reduction of GLA levels in the heart would be attributed to loss of transduced cardiomyocytes. However, since AAV transduces the liver, the decrease in GLA levels over time in serum may be attributed to the natural turnover of hepatocytes and loss of transduced cells.

The ability of [^{18}F]AGAL to interrogate the levels of GLA after gene therapy was further evaluated, by measuring the accumulated radioactivity in tissues and blood with a gamma counter. Our data showed that there was higher uptake in the organs and blood of gene-therapy-treated Fabry mice than that of control animals (Figure S4). Importantly, there was good correlation across the organs of AAV_{GLA}-treated mice, demonstrating the same rank order in [^{18}F]AGAL uptake other than the kidneys of the two animals with the lowest accumulation. This may be attributed to the competing clearance and retention processes of the tracer, because the imaging data revealed that the probe primarily underwent renal clearance and elimination via the urinary bladder, while signal emanating from the intestines may suggest a secondary elimination route in mice. This is compounded by the radiotracer's rapid kinetics after intravenous (i.v.) administration (Figures S5 and S6), and physiological processes affecting uptake and elimination by the kidneys and liver in gene-therapy-treated mice. Analyses of time-activity uptake data showed no apparent differences in [^{18}F]AGAL's cardiac uptake at 6 and 26 weeks in wild-type and AAVnull-treated mice (Figures S5E and S6E).

Confirmation of [^{18}F]AGAL's specificity toward GLA

Subsequently we determined whether the *in vivo* association of [^{18}F]AGAL to GLA is specific, to bolster its utility as a potential translational probe for the non-invasive monitoring of the enzyme's levels during gene therapy. Toward this, we used non-radioactive [^{19}F]AGAL as a homologous blocking agent, and Migalastat, a heterologous blocking agent that similarly binds to GLA's active site.[11] We imaged Fabry mice 4 weeks after AAV administration on two different days, obtaining a baseline reading on day 0 and a follow-up scan on day 3 where the blocking agent was dosed 15 min before injection of [^{18}F]AGAL. Migalastat and [^{19}F]AGAL successfully blocked binding of the radiotracer by more than 75% in the heart of the AAV_{GLA}-treated Fabry mice (Figure 3). These compounds achieved comparable reduction in the liver of AAV_{GLA}-transduced animals, suggesting that [^{18}F]AGAL specifically binds to the GLA's active site *in vivo* (Figure S7). *Ex vivo* autoradiography confirmed the *in vivo* imaging data, with the heart and liver sections of AAV_{GLA}-treated mice demonstrating elevated [^{18}F]AGAL binding (Figure 4). As these animals were dosed with blocking agents, the heart signal was lower by ~80% compared with previous autoradiographic evaluation (Figure 1B), which aligns with the reduction observed in SUV. The substantial decrease in the binding of [^{18}F]AGAL to the kidneys of animals dosed with the blocking agents

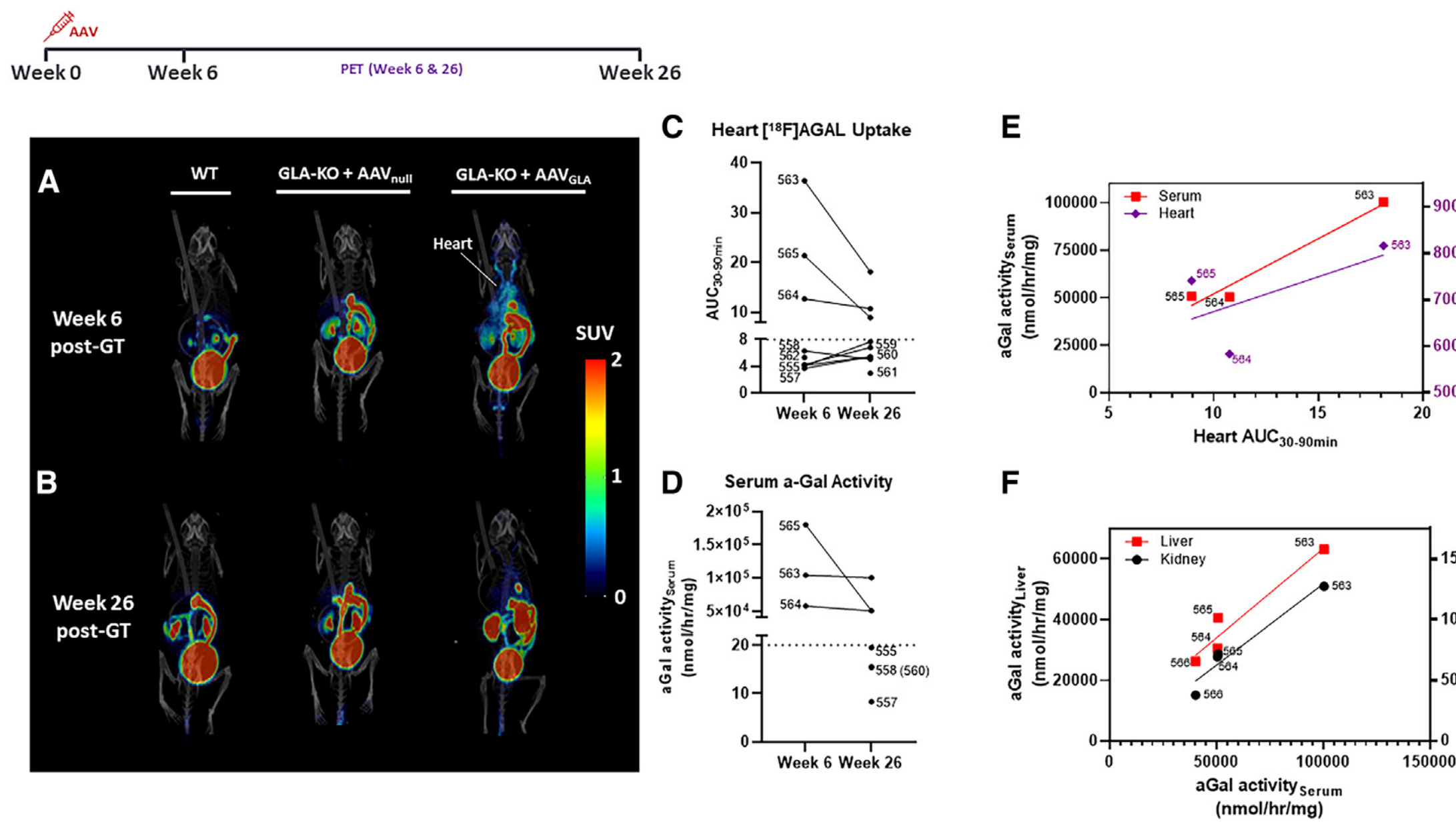

Figure 2. Longitudinal evaluation of gene therapy in a mouse model of Fabry disease using [^{18}F]AGAL and PET imaging

Coronal maximum intensity projection images of representative mice that underwent PET imaging with [^{18}F]AGAL at (A) 6 and (B) 26 weeks after AAV-based gene therapy, demonstrating increased radioactivity uptake in the heart of the AAV_{GLA}-transduced mice. (C and D) Quantification of the PET signal in the heart and determination of serum α-galactosidase A (GLA) activity, showing higher [^{18}F]AGAL uptake in the heart and serum GLA activity in the AAV_{GLA}-treated mice. Three-digit numbers correspond to the animals' identification numbers (WT: 555, 557, 558; AAV_{null}-treated: 559†, 560, 561, 562; AAV_{GLA}-treated: 563, 564, 565, 566‡). The GLA serum levels of mice 561 and 562 were below the assay's limit of quantification at weeks 6 and 26, and at week 6 for mice 555, 557, and 558. †Animal was found dead in cage, and tissue and blood could not be collected. ‡Animal did not undergo imaging. (E) The [^{18}F]AGAL heart uptake in AAV_{GLA}-treated mice correlates with GLA activity in the heart and serum. (F) The GLA activity in the liver and kidneys correlates with GLA activity in the serum indicating AAV_{GLA} achieved transduction of clearance organs. SUV, standardized uptake value; AUC, area under the time activity curve derived from standardized uptake values between 30 and 90 min scan time; WT, wild-type; GLA-KO, GLA knockout Fabry mice; AAV_{GLA}, AAV encoding human α-Gal A; AAV_{null}, AAV not encoding a therapeutic protein.

may be partly attributed to Migalastat's and [^{19}F]AGAL's renal clearance,[12] where in conjunction with the 40 times lower expression of GLA in this organ than the liver (Figure 2F) may have left limited vacant enzyme active sites for the radiotracer. Evaluation of the heart time-activity uptake curves of all animals revealed that one mouse in the homologous block cohort was likely not transduced, since on both imaging days it had comparable uptake pattern with untreated Fabry mice (Figure S8). Similarly, variability in the uptake of [^{18}F]AGAL between groups B and C at baseline (Figures 3A and 3C) was most likely attributed to differences in transduction between the two groups. This

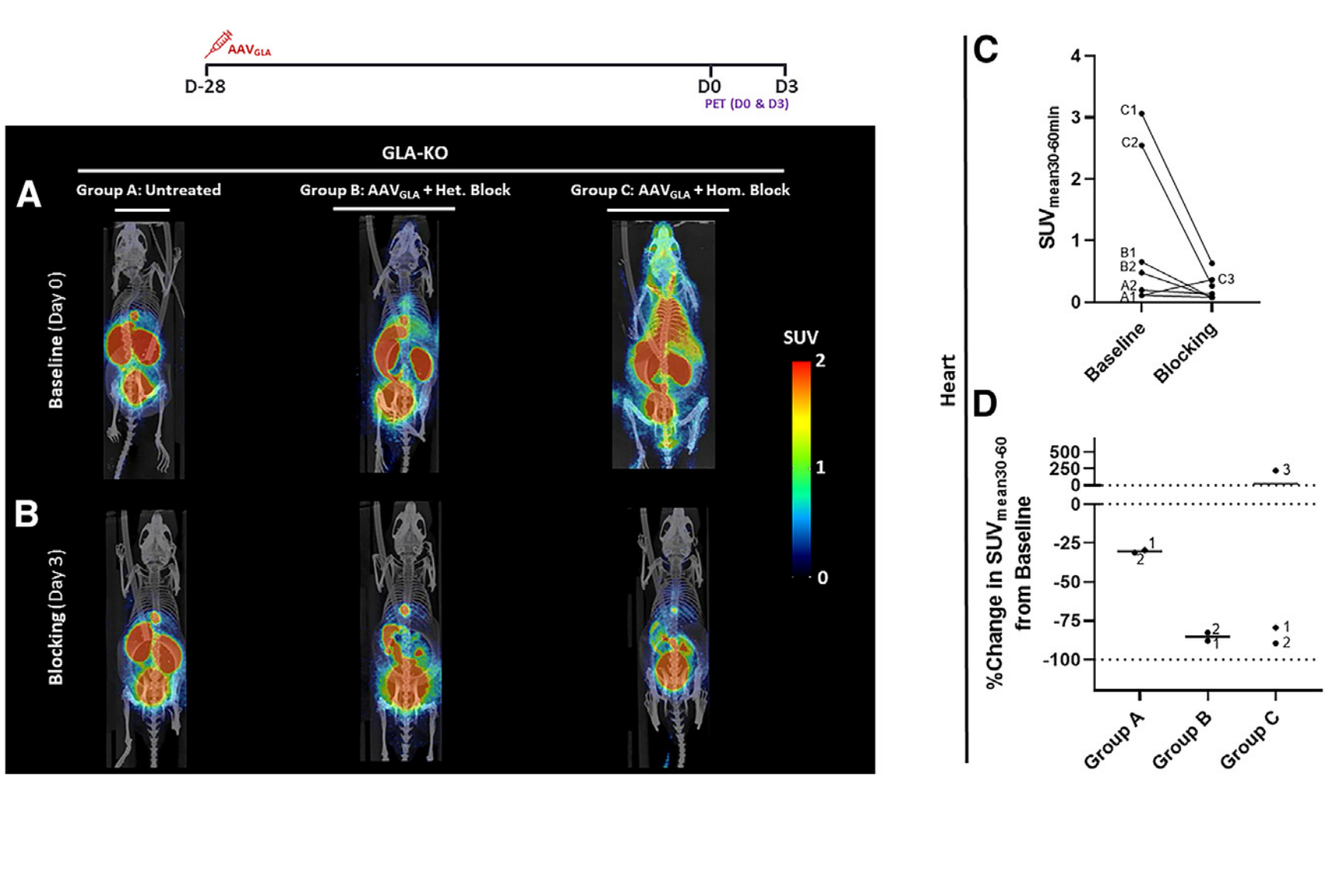

Figure 3. *In vivo* determination of the specificity of [^{18}F]AGAL binding to human GLA

Coronal maximum intensity projection images of representative AAV_{GLA}-treated Fabry mice that underwent PET imaging with [^{18}F]AGAL (A) at baseline (imaging day 0) and (B) following intravenous administration of a heterologous or homologous blocking agent at 15 min before tracer injection (imaging day 3). Heterologous block was done with Migalastat, and homologous block was achieved with [^{19}F]AGAL (Migalastat and [^{19}F]AGAL were dosed at 1 mg/kg).

(C and D) Quantification of the PET signal in the heart and determination of the changes in the tracer's heart uptake due to the blocking agent. Each datapoint depicts the corresponding animal identifiers from each group. SUV, standardized uptake value; AUC, area under the time activity curve derived from standardized uptake values between 30 and 60 min scan time; AAV_{GLA}, AAV encoding human α-Gal A; Het. and Hom. Block, heterologous and homologous blocking, respectively.

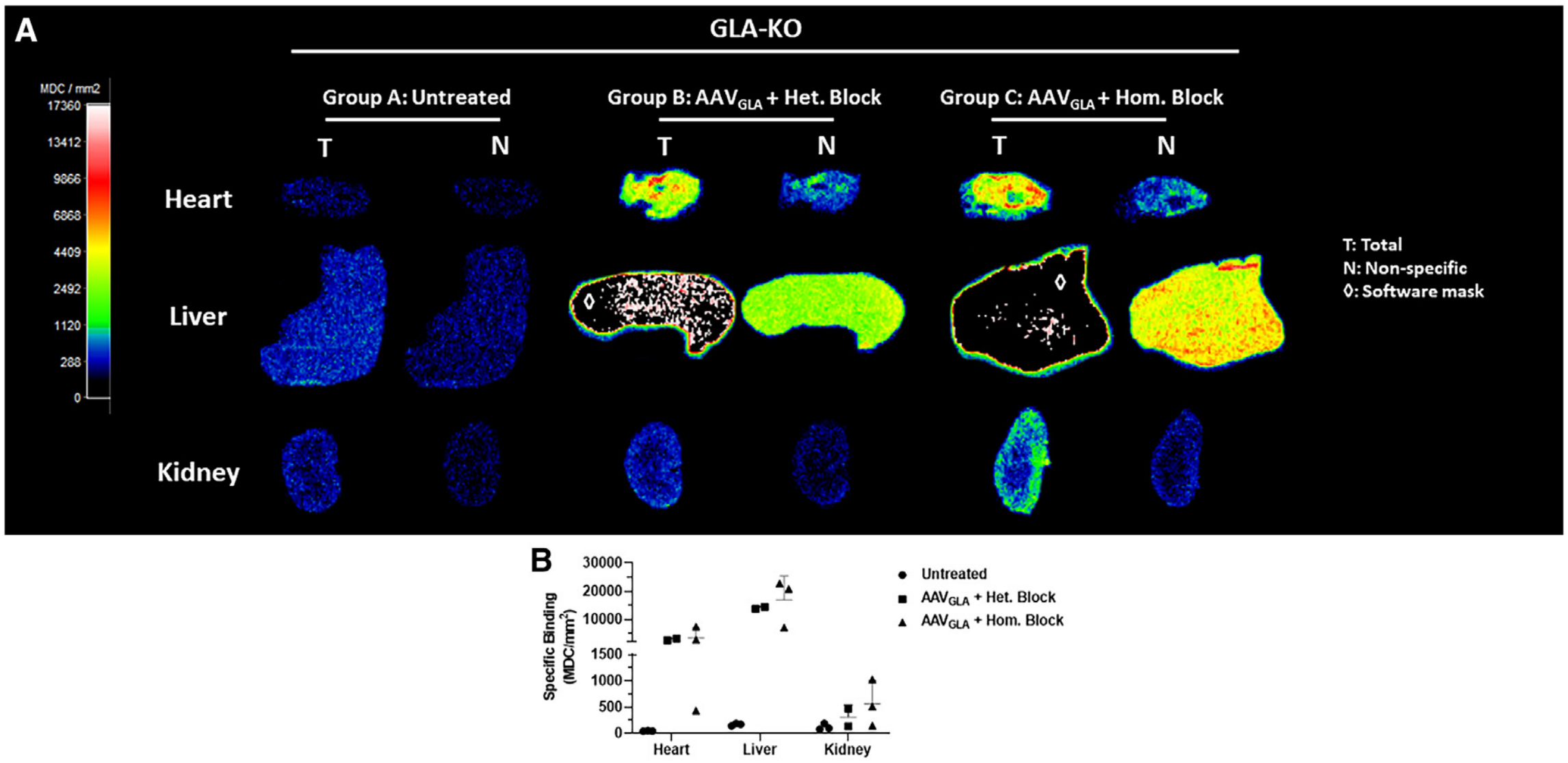

Figure 4. *Ex vivo* confirmation of [^{18}F]AGAL binding to GLA in organs of gene-therapy-treated Fabry (GLA KO) mice

(A) Representative autoradiography images of heart, liver, and kidney sections incubated with [^{18}F]AGAL at pH 5.0, indicating that the probe binds to the expressed enzyme in these organs of the AAV_{GLA}-transduced mice. (B) Quantification of the autoradiography analysis, demonstrating increased specific binding of [^{18}F]AGAL to the hearts and liver of Fabry mice transduced with AAV_{GLA}. Lower specific binding was observed in the kidney. MDC/mm^2, molecular dynamic counts per square millimeter; GLA-KO, GLA knockout Fabry mice; AAV_{GLA}, AAV encoding human GLA; Het. and Hom. Block, heterologous and homologous blocking, respectively. Software-masked area represents part of the slice where the counts reached saturation.

is supported by the data in Figures 4A and 4B, as well as Figures S1 and S2, which show that organs of some animals of group C may have been better transduced by the vector. Figures S1 and S2 also demonstrate this variability among AAV_{GLA}-treated animals. To further deduce how [^{18}F]AGAL binds to its target *in vivo*, we administered the tracer 30 min before dosing the blocking agent. PET-derived time-activity curves of the heart showed high uptake in AAV_{GLA}-treated mice, indicating that [^{18}F]AGAL likely binds covalently to GLA (Figure S9). Taken together, these studies showed that the probe specifically binds to GLA, making it a translational pharmacokinetic biomarker.

Comparison of [^{18}F]AGAL uptake in gene-therapy- vs. enzyme-replacement-treated Fabry mice

Acknowledging that a significant benefit of the treatment of monogenic diseases with gene therapy is AAV's ability to reintroduce the missing functional protein to organs of interest, we used [^{18}F]AGAL and PET imaging to compare ERT and gene therapy in Fabry disease. Toward this, we first transduced a set of GLA KO mice with AAV_{GLA} (day −28), and after 28 days we imaged them on two consecutive days with a first scan on day 0 and a follow-up scan on day 1 (schematic Figure 5). ERT was administered in a separate set of naive, non-transduced Fabry mice immediately after the imaging session of day 0 (baseline scan) and 24 h before the imaging session on day 1, to recapitulate the GLA levels observed in tissue and circulation after ERT in the clinic. Although PET imaging showed enhanced uptake of [^{18}F]AGAL in the heart of the AAV_{GLA}-treated Fabry mice (Figure 5A), there was minimal uptake of the tracer in the heart of wild-type and ERT-treated Fabry mice (Figure S10A). In line with the clinical observations that ERT does not achieve reintroduction of GLA in the heart, we saw no difference in the tracer's heart uptake before or after ERT. Small increases in [^{18}F]AGAL's uptake were observed in the liver and kidney after ERT (Figures S10B and S10C). Intriguingly, we observed uptake of [^{18}F]AGAL in the lung of gene-therapy-treated mice, but not in wild-type and ERT-receiving animals, which may be attributed to AAV-GLA-transduced mice expressing GLA in the lung or uptake of GLA via a compensatory cross-corrective mechanism, such as the liver and heart secreting GLA in circulation (Figure S10D).[5] Like our prior imaging studies described above we saw significant bladder and gallbladder uptake in all cohorts (Figures S10E and S10F), indicating that the tracer is eliminated primarily through the urinary bladder and in lower extent via biliary excretion, potentially due to [^{18}F]AGAL's size and lipophilicity. To further establish *in vivo* and *in vitro* correlations, we confirmed the *in vivo* imaging data through biochemical analyses of the hearts at the end of the study and determined that gene-therapy-treated animals had approximately 400 times more GLA than ERT animals (Figure 5B). These data agreed with immunohistochemical results that showed modest uptake of GLA by cardiomyocytes 24 h after ERT, in contrast to the wider expression the protein in the heart of gene-therapy-treated Fabry mice (Figures S11 and S12). Comparison of the rank orders of the [^{18}F]AGAL cardiac uptake vs. GLA's enzymatic activity in the heart tissue showed excellent correlation (Figure 5C), demonstrating that PET imaging can potentially provide critical information for transgene expression during Fabry gene therapy.

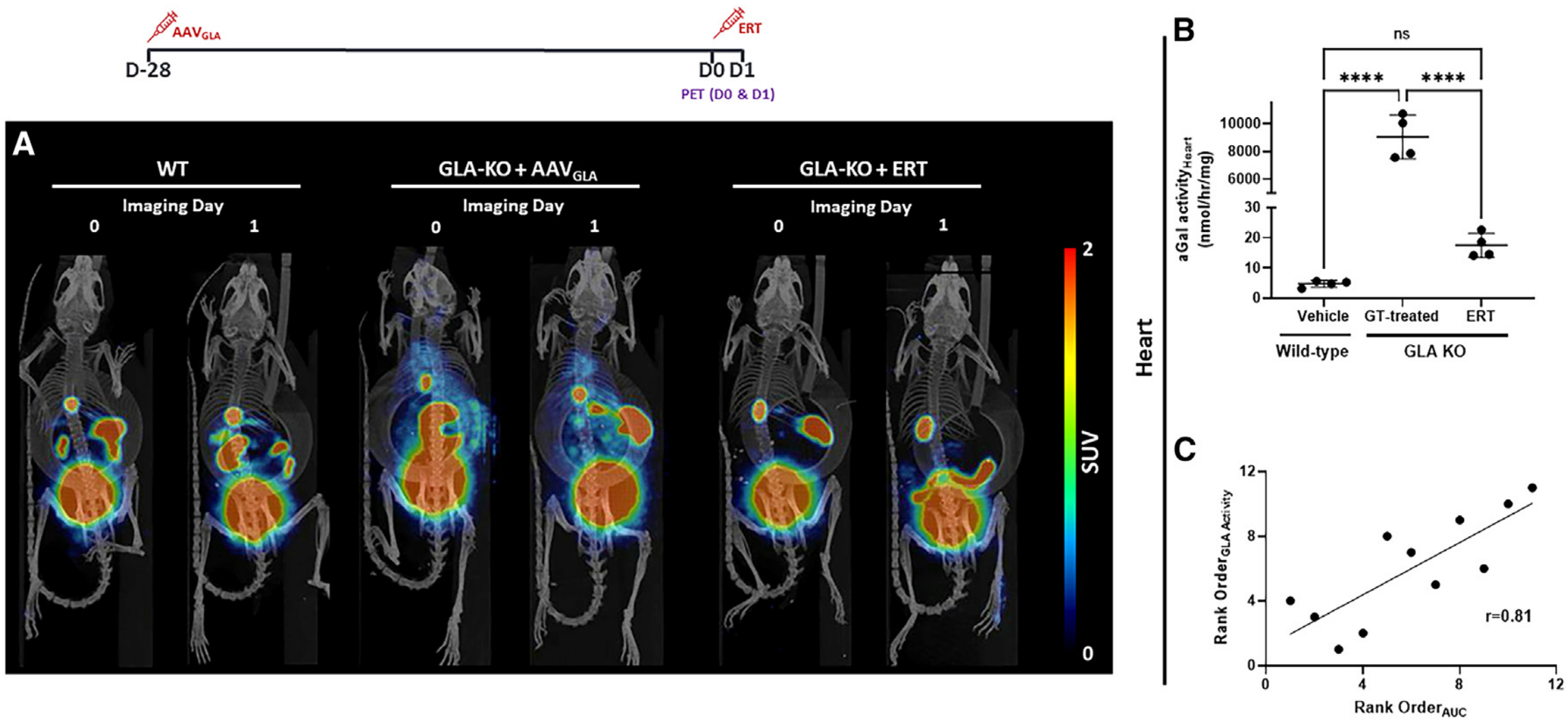

Figure 5. Comparison of gene therapy vs. enzyme replacement therapy in mice lacking GLA expression

(A) Coronal maximum intensity projection images of representative Fabry mice that received AAV_{GLA} gene therapy or enzyme replacement therapy. PET [^{18}F]AGAL imaging was performed on two imaging days (day 0: 28 days after gene therapy; day 1: 24 h after enzyme replacement therapy) showing higher radioactivity uptake in the heart of mice with AAV_{GLA} gene therapy than enzyme replacement therapy. (B) *Ex vivo* determination of GLA in the heart, showing higher enzyme activity levels in AAV_{GLA}-treated mice. Statistical significance based on one-way ANOVA. ****$p < 0.0001$; ns, not statistically significant. (C) Correlation plot of the rank order of the [^{18}F]AGAL *in vivo* heart uptake vs. the *ex vivo* cardiac α-Gal A activity. r, Pearson correlation coefficient. SUV, standardized uptake value; WT, wild-type; AAV_{GLA}, AAV encoding human GLA; ERT, enzyme replacement therapy; AUC, area under the time activity curve derived from standardized uptake values between 30 and 60 min scan time.

Preclinical evaluation to support [^{18}F]AGAL's clinical translation

We first evaluated the tracer's retest variability in AAV_{GLA}-treated mice that were imaged on two consecutive days. [^{18}F]AGAL exhibited a retest variability of less than 10% in the heart and approximately 10% in the liver (Figure S13), indicating that it is an excellent PET tracer.[13] Lastly, we evaluated our radiotracer [^{18}F]AGAL in a healthy NHP to obtain pharmacokinetics and radiation dosimetry estimates in a higher species for potential translation to humans. Like its uptake in wild-type and untreated Fabry mice, [^{18}F]AGAL showed high uptake in the kidneys and urinary bladder of the NHP, demonstrating elimination predominantly via the urinary pathway, with a small hepatobiliary component (Figure 6). Time-activity uptake curves further confirmed the quick clearance of the tracer from the kidneys to the urinary bladder. The liver and heart were the non-elimination organs with the highest uptake with relatively slower washout kinetics. Lower uptake with reversible kinetics and moderate washout was observed in the testes and lumbar vertebrae (marrow) (Figure S14). The three highest kinetic values for source organs were found for urinary bladder, small intestine, and kidneys (Table 1). The estimated whole-body effective dose (ED ICRP-103) was 0.0256 mSv/MBq, in line with other ^{18}F-labeled PET tracers.[14] The urinary bladder wall received the highest organ dose (4.03E−01 mSv/MBq) based on a urinary bladder voiding model with 2-h voiding interval (Table 2). Although the highest dose organ is the urinary bladder limiting the number of [^{18}F]AGAL injections to two per year at 185 MBq (5 mCi/injection) or five per year at 74 MBq (2 mCi/injection) according to 21 CFR 361.1, voiding the bladder immediately after the scan may mitigate radiation exposure.

DISCUSSION

The treatment of rare monogenic diseases, such as Fabry disease, with gene therapy could provide long-term AAV-mediated production of the therapeutic protein, overcoming the limitations of i.v. ERT where organs, such as the heart, do not receive adequate levels of the corresponding protein. Systemically dosed AAV therapeutics that have been innovatively engineered, including the transgene's regulatory elements and capsid's tropism, have the potential to transduce deep tissues and locally produce the therapeutic protein. In addition, they may also facilitate cross-correction of other organs through the production of the therapeutic by a depot organ, which may then release this protein in circulation for uptake by a distal site, while conferring immunotolerance.[15] Therefore, having a non-invasive biomarker that can directly interrogate the levels of target protein by these novel therapeutics in whole body including deep tissues is critical.

Herein, we have demonstrated that the levels of active GLA after AAV-mediated transduction can be detected *in vivo* in deep tissues such as the heart using a novel PET imaging tracer [^{18}F]AGAL developed for Fabry disease. To our knowledge, this is the first report of any PET tracer directly reporting the magnitude, location, and durable expression of an active lysosomal enzyme for a lysosomal storage disease. Our studies revealed that [^{18}F]AGAL has a high affinity to GLA, through its ability to covalently bind to the enzyme's active site at pH 5.0, which is comparable with the acidified milieu of lysosomes, which is the intracellular site of Gb_3 accumulation in Fabry disease. We showed that the radiotracer could longitudinally evaluate gene therapy in Fabry mice with high radioactive uptake in heart as early as

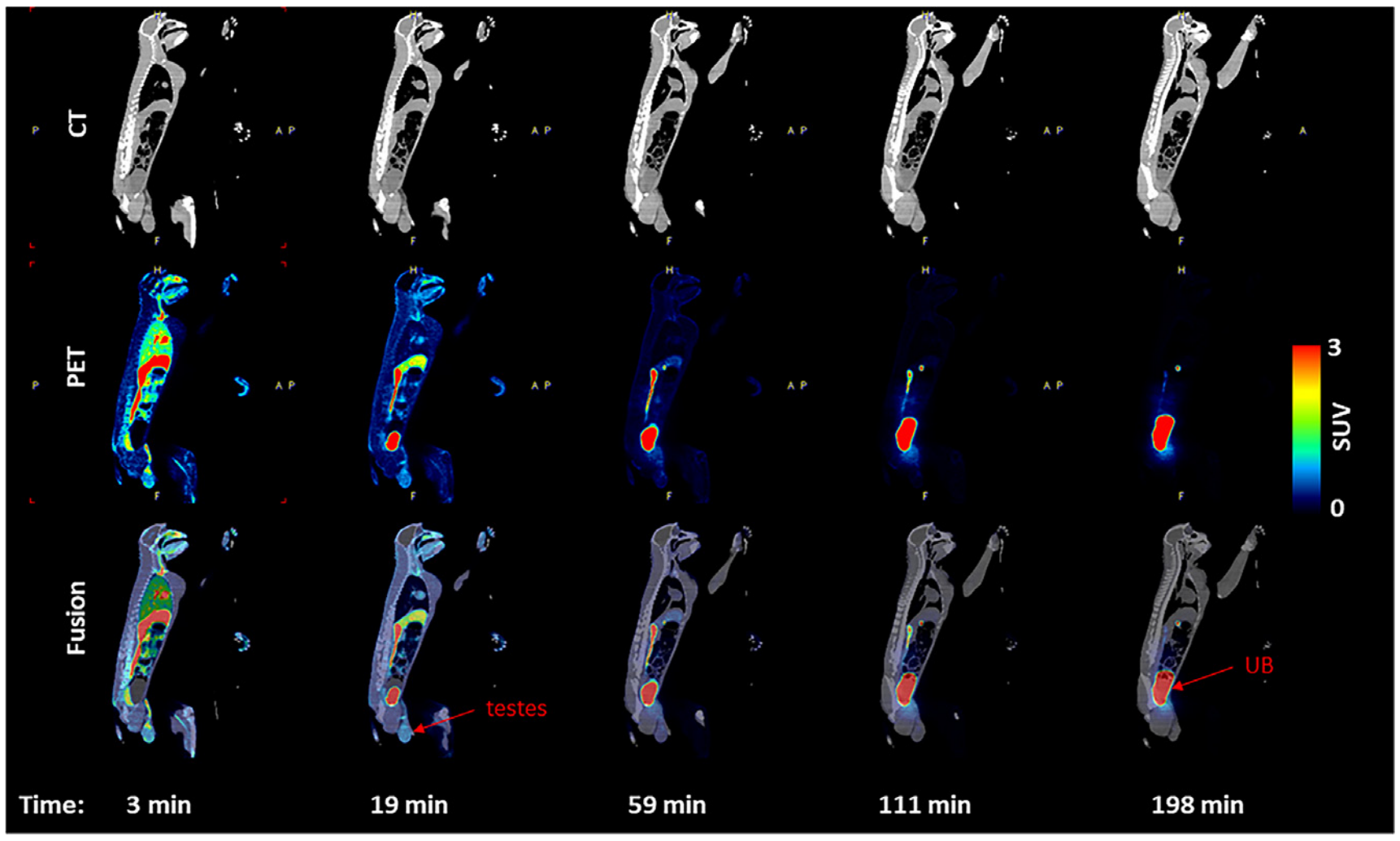

Figure 6. PET-based monitoring of the distribution of [^{18}F]AGAL in a healthy non-human primate

Sagittal CT (top row), PET maximum intensity projection (middle row), and PET/CT fusion (bottom row) images of a male monkey, taken at different times after [^{18}F]AGAL administration. The tracer undergoes renal clearance and elimination via the urinary bladder (UB). SUV, standardized uptake value; Fusion, CT and PET images merge.

6 weeks and up to 26 weeks after transduction, reflecting AAV's robust and long-lasting transgene expression in the heart. PET imaging with [^{18}F]AGAL also showed good correlation with *ex vivo* biochemical and autoradiographic assays as well as correspondence with histological staining including gene therapy signal outperforming ERT signal in target tissues. [^{18}F]AGAL exhibited excellent retest variability, and dosimetry calculations based on NHP imaging showed that it has an estimated whole-body effective dose comparable with other ^{18}F-labeled tracers that are used in the clinic.

Current diagnostic workup and follow-up monitoring of Fabry disease consist of extensive evaluations of genetic, clinical, and biochemical markers for renal, cardiovascular, and metabolic functions, including evaluation of GLA activity and globotriaosylsphingosine (LysoGb3) in blood samples.[16] Although quantification of the levels of Gb_3 in the urine showed dose-dependent response to ERT,[17,18] its clinical relevance has not been further investigated. Instead, it is recommended to monitor the plasma levels of $LysoGb_3$ as a potential disease activity biomarker.[19–22] However, the major limitation of these biomarkers is that they do not inform about disease burden in each organ, but rather provide an overall body-wide metric. In a clinical trial setting, organs, such as the kidney and liver, may undergo biopsy to provide proximal information for Gb_3 accumulation, while performing renal biopsies in pediatric Fabry is still contested within the community of treating physicians.[23] Thus, having a non-invasive imaging tool that monitors the levels of active GLA in organs such as the heart, particularly for novel single one-and-done therapies such as gene therapies, can provide clinical trialists with the means to longitudinally assess a biomarker that is most proximal in the disease mechanistic pathway. Such a proximal imaging tool in combination with the existing set of distal PD efficacy and clinical biomarkers can also facilitate treating physicians to better manage the symptoms of the disease.

Table 1. [^{18}F]AGAL kinetic values (or total number of disintegrations) in source organs of a male rhesus macaque

Source organ	Kinetic value[a] (MBq-h/MBq)
Gallbladder cont.	1.43E−03
Left colon	1.72E−02
Small intestine	1.76E−01
Right colon	9.54E−02
Rectum	3.10E−03
Heart wall	1.99E−02
Kidneys	1.18E−01
Liver	2.22E−02
Lungs	1.57E−02
Red marrow	6.56E−03
Testes	5.15E−04
Urinary bladder cont.[b]	8.22E−01
Remainder	2.72E−01

MBq, megabecquerel; MBq-h, Megabecquerel-hour.
[a]Values were scaled based on differences in organ and body weights between human and rhesus monkey.
[b]Urinary bladder was modeled with a voiding interval of 2 h.

In this study, we used a fluorine-18-labeled AGAL probe in a two-step radiosynthesis described previously.[8] [^{18}F]AGAL's tracer kinetics and tissue uptake, particularly tracer retention in AAV_{GLA}-transduced mouse heart (high), liver, and kidneys (moderate to low) with a rapid biodistribution and clearance from non-gene-therapy-treated organs in mouse and normal NHP provides a favorable profile, plausibly allowing patient imaging within an hour of tracer administration. The moderate to low signal differences in liver and kidneys could still be due to low level of non-specific background signal that could be present in those metabolically active organs. However, the radiolabeling approach we used allows the potential substitution of fluorine-18 with other radionuclides with longer radioactivity half-lives, such as copper-64 ($T_{1/2}$ = 12.7 h) and zirconium-89 ($T_{1/2}$ = 78.4 h). The use of radiometals with longer half-lives may provide the opportunity to better image hepatic and renal expression of GLA at later time points, since the radiotracer would have been eliminated by the urinary bladder minimizing potential partial-volume effects as well as any potential radiometabolite contamination. To accommodate these radiolabeling alternatives, the strain-promoted azide-alkyne click

Table 2. Radiation dosimetry estimates for [^{18}F]AGAL in an adult human model (ICRP-89)

Target organ	Radiation dose (mSv/MBq)
Adrenals	1.30E−02
Brain	1.25E−03
Esophagus	3.23E−03
Eyes	1.28E−03
Gallbladder wall	1.10E−02
Left colon	2.78E−02
Small intestine	5.50E−02
Stomach wall	4.09E−03
Right colon	6.46E−02
Rectum	2.58E−02
Heart wall	1.26E−02
Kidneys	7.35E−02
Liver	6.87E−03
Lungs	4.01E−03
Pancreas	8.42E−03
Prostate	3.02E−02
Salivary glands	1.47E−03
Red marrow	6.08E−03
Osteogenic cells	4.12E−03
Spleen	5.60E−03
Testes	9.51E−03
Thymus	2.64E−03
Thyroid	1.78E−03
Urinary bladder wall	4.03E−01
Total body	6.94E−03
ED (ICRP-103)	2.56E−02

mSv/MBq, millisievert per megabecquerel.

chemistry or the inverse electron demand Diels-Alder reaction may be adopted to introduce a radiometal chelator without compromising radiochemical purity and yield.[24] Our present approach indicates that such bioconjugation strategies will likely not affect the probe's affinity to GLA, because the coupling will take place further away from the aziridine moiety that interacts with the enzyme's active site.

Use of PET imaging for gene and cell therapy monitoring in drug development space has recently gained traction largely due to efforts of indirect reporter gene imaging and direct imaging of transgene activity by PET tracers that are established in the clinic.[25–27] Effective reporter gene imaging requires inclusion of a reporter gene along with the therapeutic gene within the limits of gene construct size. The ensuing reporter protein levels quantified by imaging need to well correlate with the therapeutic protein levels at the target site, and besides the reporter protein by itself should be non-immunogenic with low background expression levels in the target tissues.[28] With persistent advances in genetic engineering and PET imaging probes, few reporter gene imaging methods are assessed robustly in preclinical space and have transitioned to first-in-human studies, particularly for oncology indications.[25,29] In contrast to reporter gene imaging, reports on direct transgene protein imaging are limited. Particularly in clinical trial monitoring there has been application of imaging in aromatic L-amino acid decarboxylase (AADC) deficiency, wherein AADC activity following AAV-mediated expression of the enzyme is imaged by fluoro-dopa PET scans in the brain.[26,30] Our [^{18}F]AGAL PET tracer offers a similar paradigm of assessing the therapeutic gene expression and activity of GLA in patients with Fabry disease treated with novel AAV-mediated or other potentially superior supraphysiological transgene expression systems targeted for heart and/or liver. Considering there was a small increase in tracer uptake in ERT-receiving mice compared with wild-type mice, [^{18}F]AGAL PET could also be applied to monitor ERT-driven tissue levels of GLA as there are improved ERT systems with potentially better tissue penetration being tested in clinical trials that may be approved for Fabry disease.[31] Considering that [^{18}F]AGAL specifically binds to GLA at the low pH conditions of the intracellular lysosomal milieu (Figure 1A), similar to the way GLA binds to its canonical substrate, the presence of GLA in the extracellular compartment (as in the plasma) following ERT or potential secretion from AAV_{GLA}-transduced organs will not confound the PET signal. This is because [^{18}F]AGAL is least likely to bind to GLA in the neutral pH of the blood. This provides a unique advantage for [^{18}F]AGAL to track the active enzyme levels in the intracellular compartment of the target organs.

Engineering advancements have led to the introduction of new technologies in the clinic, including combined PET/magnetic resonance imaging (PET/MRI) systems and total-body PET/CT systems.[32] These newer systems have multiple advantages over the traditional PET or PET/CT systems. PET/MRI systems combine the molecular and functional information from PET with the high-resolution anatomical data from an MRI, allowing comprehensive evaluations in a single imaging session. Total-body PET/CT systems provide high detection sensitivity (>40-fold) and spatiotemporally superior information resulting in increased signal-to-noise ratio, reduction in imaging time, as well as significant reduction in radiation dose potentially allowing scans in pediatric subjects.[33] Since gene therapy trials are expected to occur in highly specialized tertiary care settings in a limited number of subjects, it is possible to make use of these advanced systems and infrastructure to deliver most effective trial readouts.

Our preclinical evaluation of [^{18}F]AGAL in Fabry mice has some limitations. First, the imaging studies were performed at a single dose of AAV_{GLA} (2.5×10^{11} vg/kg), which corresponds to the highest dose of the vector that conferred robust efficacy in a mouse model of Fabry disease. Since PET imaging is regarded as a sensitive and quantitative technique, where very low mass doses of the probe are used (typically microdoses resulting in pico- or femtomolar target concentrations) to bind to targets present at high nanomolar concentrations, it is possible to observe linear changes of tracer uptake (i.e., lower or

even higher than in this study) in a study with different AAV_{GLA} dosing levels. Such a study may enable the use of the PET tracer in the clinic for AAV dose selection, development of a PK/PD model using PET-derived therapeutic enzyme concentrations at the target site of action, and effective longitudinal monitoring of gene therapy. Second, due to the logistical complexity of conducting PET studies, a small number of mice (n = 3–4 per group) were used in our evaluation for each treatment group. Despite the small sample size, the high sensitivity of PET yielded a signal magnitude in almost all studies that demonstrated a statistically significant difference between test and control or wild-type animals. This suggests that the PET tracer could effectively quantify its target in the target tissue, particularly the heart, which is an organ of major importance in Fabry disease. Third, because our *in vivo* tracer validation was performed only in mice, the PET signal quantitation in this study was limited to SUV or AUC, which are semiquantitative metrics. Typically for improved accuracy of PET tracer readouts, full quantitation is obtained by dynamic PET studies with metabolite-corrected input function that requires collection of arterial blood samples followed by PET pharmacokinetic modeling, to derive the tracer's volumes of distribution (V_T) or influx rate constant (K_i). These dynamic PET studies are performed in larger animals, such as rats and NHPs. Fourth, the biodistribution and radiation dosimetry of [^{18}F]AGAL was performed in a single healthy NHP, and as a result there is lack of variability in dosimetry estimates for humans. Thus, further evaluation of [^{18}F]AGAL in a larger cohort of NHPs with whole-body dynamic PET will enable this tracer to advance for first-in-human studies. Despite these limitations, this study provides a comprehensive proof-of-concept dataset for potential use of [^{18}F]AGAL to detect the levels of active GLA in target tissues of Fabry disease patients, and to monitor treatment effect in clinical therapeutic trials. In general, it also provides a solid framework to evaluate any new PET tracer intended for the direct imaging of transgene expression in a specific organ following gene or enzyme replacement therapies.

In summary, our data demonstrate that [^{18}F]AGAL is a promising PET tracer for the detection and longitudinal tracking of GLA expression in the heart following gene therapy in a mouse model of Fabry disease. Imaging findings showed good correlation with confirmatory *ex vivo* biochemical assays. This probe has an attractive translational profile due to its specificity, mechanism of target binding, and kinetics, making it suitable for application as a pharmacokinetic/pharmacodynamic biomarker in gene therapy trials of patients with Fabry disease. Like other approved ^{18}F-labeled radiotracers, it has an acceptable dosimetry that accommodates imaging for several times in a year if needed, thus making [^{18}F]AGAL a clinically translatable biomarker for the long-term monitoring of Fabry gene therapy.

MATERIALS AND METHODS

[^{18}F]AGAL synthesis

Multi-step syntheses were carried out to prepare the azido AGAL precursor and the fluoroalkyl reference standard compound ([^{19}F]AGAL) at MedChem Imaging (Boston, MA), following literature procedures with slight changes to accommodate the reactions to 100 and 50 mg, respectively.[34–36] All compounds were characterized by ^{1}H NMR spectroscopy and LC-MS for the structure and purity. Radiolabeling of the azido AGAL precursor was performed at the Molecular Cancer Imaging Facility of the Dana-Farber Cancer Institute (Boston, MA) as described previously.[8] In brief the azido AGAL precursor reacted with [^{18}F]-labeled pentyne in the presence of copper (II) sulfate (Sigma-Aldrich, St. Louis, MO) and TBTA (Combi-Blocks, San Diego, CA), using the GE FX2 N radiosynthesis module. [^{18}F]AGAL was synthesized and characterized as reported previously,[8] with radiochemical purity greater than 95%, and specific activity greater than 2,700 mCi/μmol.

Recombinant AAV information

Recombinant AAV_{GLA} and AAV_{null} were prepared and characterized using established internal procedures (Takeda), as described recently.[37] AAV_{GLA} encoded the human α-galactosidase gene (*GLA*; NM_000169), and AAV_{null} comprised a non-coding DNA sequence. The genome titer was determined by ddPCR, the purity was assessed by either SDS-PAGE followed by silver staining or CE-SDS to visualize VP1, VP2, and VP3, and the absence of endotoxin was assessed by a limulus amebocyte lysate assay (Endosafe). The aliquoted AAVs were stored at –80°C until further use.

Animal studies and tissue processing

The Fabry mouse colony used in this study was maintained at Taconic Biosciences (Boston, MA). The studies conducted followed Animal Welfare and Institutional Animal Care and Use Committee Review guidelines and were approved by the Institutional Animal Care and Use Committee (IACUC). Male mice were injected with AAV test articles (2.5×10^{11} vg/kg) intravenously at the age 8–12 weeks, and were euthanized at the indicated time for each study. Blood was collected via cardiac puncture and processed to separate serum. Mice were transcardially perfused with PBS for 4–5 min or until the tissues were cleared of blood prior to collection. Tissues and serum for analytical endpoints were harvested and snap-frozen, followed by cryopreservation at −80°C. Tissues were prepared for α-Gal activity through homogenization using the Precellys24 system (Bertin Instruments, France) with appropriately sized beads, tubes, and lysis buffer volumes (10 mM HEPES, 0.5% Triton X-100, 1× protease inhibitors). Supernatant from tissues was collected and evaluated by the α-Gal enzyme activity assay.

GLA enzyme activity

GLA activity was determined using the 4-MU-α-gal fluorescent substrate. In brief, a biological sample (serum or tissue homogenate) of 2 μL was incubated with 15 μL 4-MU-α-gal substrate solution (M65400, Research Products International Company, Mt. Prospect, IL) with α-galactosidase B inhibitor (N-acetyl-D-galactosamine A-2795, Sigma-Aldrich Chemicals, St. Louis, MO) at 37°C for 60 min. The enzymatic reaction was stopped by addition of 200 μL glycine carbonate stop solution (pH 10.7). The fluorescence of the 4-MU product at 465 nm was measured followed excitation at 360 nm, using a fluorescence plate reader. The concentrations of 4-MU in testing samples were calculated from the 4 MU calibration

curve in the same plate. Serum and tissue GLA activity was normalized to the volume of serum, or tissue's total protein concentration that was determined by the BCA assay and expressed as nmol of 4-MU substrate produced per hour per mL of serum or mg of total protein.

Small animal PET/CT imaging

PET imaging with [^{18}F]AGAL and CT were performed on dedicated small-animal PET scanners (Siemens Multimodality Inveon, Siemens Medical Solutions USA, or Molecubes β-Cube and X-Cube, Molecubes). The mice were anesthetized using isoflurane/medical air inhalation prior to the radiotracer injection and throughout the scan duration. During anesthesia, warming was provided to maintain healthy core body temperature. [^{18}F]AGAL was administered as a bolus i.v. injection via the lateral tail vein (1.85–7.40 MBq/50–200 μCi). Dynamic scans were acquired in list mode format over 90 min, and the collected data were then sorted into 0.5-mm sinogram bins for image reconstruction using FORE/3D-OSEM-MAP. Following the PET acquisition, a low dose CT scan was acquired (80 kVp, 0.5 mA) for anatomical reference and to provide guidance for the delineation of selected tissues regions of interest (ROIs). Wherever indicated, static whole-body scans were performed 30 min after [^{18}F]AGAL administration, with the energy window spanning 420–580 keV via continuous-bed motion acquisition. Migalastat (Sigma-Aldrich) and [^{19}F]AGAL (MedChem Imaging) were used, respectively, for the heterologous and homologous block. They were administered as a bolus i.v. injection at 1 mg/kg either 15 min before (blocking study) or 30 min after (chase study) the dosing of the [^{18}F]AGAL. Animals receiving ERT were dosed i.v. with 1 mg/kg recombinant human α-Gal A (Takeda) 24 h before PET imaging. For dynamic scans, PET list mode data were reconstructed using ordered subset expectation maximization into a series of 36 frames (4×15, 5×60, and 27×120 s), with corrections for attenuation, randoms, scatters, dead time, radioactive decay, and detector normalization. For static scans, PET data were reconstructed in a single frame using the same algorithm. Reconstructed images had dimensions of $192 \times 192 \times 384$, pixel sizes of $0.4 \times 0.4 \times 0.4$ mm, and units of kBq/cc.

PET image data analysis

PMOD 4.2 software (PMOD Technologies LLC) was used to visualize and quantify the PET data. Volumes of interest were manually drawn over the heart, liver, lung, kidney, femur, muscle (quadriceps), gallbladder, and bladder. Time activity curves were generated for dynamic studies and uptake in each tissue was expressed in units of SUV based on injected activity dose and most recently recorded animal weights.

Immunohistochemistry

The dissected tissue samples were quickly rinsed with PBS and trimmed to <0.5 mm in thickness, and then immediately fixed in 10% neutral buffered formalin (catalog no. SF100-20, Fisher Chemical, Thermo Fisher Scientific) with the volume ratio of tissue-to-fixative (1:20) on an orbital shaker at room temperature for 48 h. Five micrometer tissue sections were collected for immunohistochemistry assay, which was performed on an automated Leica Bond system (Leica Biosystems, Wetzlar, Germany) using the Bond Polymer Refine Detection kit (catalog no. DS9800, Leica Biosystems). The section was pre-treated for 20 min using heat-mediated antigen retrieval with Bond epitope retrieval solution 1 (pH 6.0, catalog no. AR9961, Leica Biosystems), followed by incubation with the α-Gal antibody (4.9 μg/mL, Lot SH006, Takeda) for 30 min at room temperature and detected using horseradish peroxidase-conjugated compact polymer system. 3,3′-Diaminobenzidine was used as the chromogen, and the sections were then counterstained with hematoxylin. The stained slides were dehydrated and mounted with Cytoseal*60 Mounting Medium (catalog no. 8310, Thermo Fisher Scientific) and coverslips using a Leica CV5030 Fully Automated Glass Coverslipper. All stained slides were scanned with the Leica AT2 Scanner and viewed and analyzed with the HALO Link Image Management System (Indica Labs).

Autoradiography

Following perfusion, the heart, liver, and kidneys were resected and subsequently frozen for cryosectioning. The tissue sections were prepared by sectioning the fresh-frozen tissues at a thickness of 10 μm (Leica CM3050S, Germany) and thaw mounting on glass slides (Superfrost Plus Gold, Fisher Scientific), followed by storage at −80°C for later use. Prior to autoradiography, the tissue sections were acclimated to room temperature. To evaluate specific binding in the presence of acidic or physiological pH, [^{18}F]AGAL was prepared by diluting stock into McIlvaine buffer (0.15 M citrate-phosphate buffer) (pH 5.0) or 50 mM Tris-HCl in 0.9% saline (pH 7.45). In initial assay optimization studies 35 nM of [^{18}F]AGAL was used, and all other studies utilized 3 nM of [^{18}F]AGAL. Non-specific binding was defined by incubation of tissue slides with or without 1 mM [^{19}F]AGAL.[38] An equivalent volume of 100% DMSO was added (<0.1% final amount) to the incubation bath defining total binding. Similarly, for saturation analysis, increasing concentrations of [^{18}F]AGAL (0.5–185.6 nM) were added to McIlvaine buffer (pH 5.0) and split between two troughs per concentration. Tissues were then incubated in the presence or absence of [^{19}F]AGAL for 60 min at 23°C. Reactions were terminated by washing in two changes of cold saline (4°C) for 3 min each followed by 10 s in cold deionized water. Slides were rapidly dried under a cool airstream. Following exposure to phosphor screens (BAS-IP TR2040E; Cytiva, Marlborough, MA) for 30–60 min, digital images were generated by scanning at 200 mm pixel size using an Amersham Typhoon 5 (Cytiva). ROI analysis was performed using MCID 7.1 (Interfocus Imaging, Cambridge, UK). Data are reported in percent specific binding or molecular density calibration units/mm^2 (MDC/mm^2) and graphically analyzed using GraphPad Prism 9.2.0 (GraphPad Software, San Diego, CA). To estimate K_D and B_{max}, we first calculated the specific binding using the formula (total signal – signal due to non-specific binding = signal due to specific binding), and then fit the resulting data to the non-linear regression equation $y = B_{max} \times x/(K_D + x)$ for one-site specific binding, with the specific binding plotted on the y axis and the corresponding [^{18}F]AGAL concentration on the x axis.

NHP PET study and dosimetry

This study was conducted in full compliance with Yale University's IACUC policies and procedures, which follow the recommendations of The Guide for the Care and Use of Laboratory Animals. One non-naive male rhesus monkey (*Macaca mulatta*, 11.3 kg, 7.4 years old) was used in this study. Before assignment to the study, the animal was examined by a veterinarian and found to be healthy. The animal fasted overnight prior to the imaging session. At least 2 h prior to radiotracer administration, the animal was induced with Alfaxan 2 mg/kg intramuscularly (i.m.) (Jurox, Parsippany, NJ), dexmedetomidine 0.1 mg/kg i.m. (Covetrus, Portland, ME), and midazolam 0.3 mg/kg i.m. (Covetrus). Once induced, the animal was placed on isoflurane (Covetrus) using a mask, and then dexamethasone 0.5 mg/kg IM (Bimeda, Oakbrook, IL) was administered to prevent nausea and emesis during recovery, as well as to control any pain or inflammation, as part of standard of care. Before intubation, lidocaine, 1% (Covetrus), was applied topically over the larynx to prevent spasm. Shortly after intubation, Antisedan 0.1 mg/kg i.m. (Generic) was given to partially reverse the effects of dexmedetomidine. Sedation was maintained with 1.7%–2.1% isoflurane administered through a rebreathing circuit, along with oxygen (1.5 L), to the end of the PET scan. The animal received Lactated Ringer's Solution plus 5% dextrose at 4–6 mL/kg/h i.v. (B. Braun, Bethlehem, PA). Body temperature was maintained at 37.2°C–37.9°C using a heated water blanket. Dynamic whole-body (crown-to-knee) PET imaging was conducted over ∼250 min immediately following injection of [^{18}F]AGAL (6.81 mCi; 252.07 MBq). The radiotracer was administered in a single i.v. dose as a bolus over 3 min using an infusion pump in a volume of 9.7 mL. PET and associated CT images were generated using a Biograph Vision 600 (Siemens) camera. Prior to the radiotracer injection, a CT scan was performed for attenuation correction and anatomical identification. Immediately following injection of the radiotracer, dynamic emission data were collected using continuous bed motion acquisition for ∼250 min and binned into a series of 24 dynamic 3D PET image volumes. The dynamic series were subsequently reconstructed using a time-of-flight with point spread function algorithm with standard corrections for detector normalization, deadtime, random events, scatter, attenuation, and radioactive decay corrections. Reconstructed PET images were transferred to a specialized workstation and analyzed using the image processing PMOD software package v.3.802 (PMOD Technologies, Zurich, Switzerland). The following organs were considered as source organs for the purpose of dosimetry calculations: heart, liver, gallbladder, kidneys, intestines, urinary bladder, lumbar vertebrae, lungs, and testes. The number of disintegrations per unit activity administered in each source organ (i.e., kinetic values), τ, were computed from the area under the non-decay corrected time-activity curves via the trapezoid method. Area under the curve from end of imaging to infinity was computed with the assumption of physical decay only following the last imaging time point. The kinetic values were converted to adult human values by using conversion factors based on differences in organ and body weights between the two species. ICRP 100 HAT GI tract model was used to estimate the kinetic values in the intestinal segments, assuming the activity enters the GI tract via small intestine, with no reabsorption. The intestinal decay corrected time-activity curve was used for estimation of the fraction of the radioactivity entering the intestine during the imaging period. To conservatively estimate the whole-body red marrow content, the measured lumbar marrow was multiplied by 10/4 based on previously reported values of red marrow mass in the vertebrae of rhesus and human subjects. OLINDA v.2.2 software (Hermes Medical Solutions) was used to estimate the organ and whole-body radiation absorbed doses. OLINDA performs internal dose calculations based on MIRD and Radiation Dose Assessment Resource methodology. The ICRP-89 adult male (73 kg) model was used to calculate the s-factors. All other MIRD assumptions about the homogeneity of source organ distribution were employed. The effective doses were computed with biological tissue-weighting factors defined in the ICRP Publication 103 (2007).

DATA AND CODE AVAILABILITY

The data that support the findings of this study are available from the corresponding author, upon reasonable request.

ACKNOWLEDGMENTS

This research was funded by Takeda Pharmaceutical Company Limited and received no external funding. The authors would like to thank Kathleen Palmieri (Takeda), the Comparative Medicine group (Takeda), and the MedChem Imaging team for expert technical assistance.

AUTHOR CONTRIBUTIONS

C.K., T.G.L., C.T.W., S.N., W.R., and R.I. conceptualized the project and aims. S.H.A., A.P.B., and P.McQ. developed the radiosynthesis method and production of the PET tracer. M.D. contributed to the design, production, and formulation of AAVs. T.T., L.M.C., and Q.-D.N. performed *in vivo* studies. A.K., P.S., L.C., W.R., and S.M. performed *in vitro* and *ex vivo* assays. S.Y. conducted histopathological analysis. Y.P. analyzed rodent PET imaging data. C.C.C. and A.L.A. conducted the NHP PET study, the NHP image analysis, and dosimetry. C.K. and T.G.L. analyzed data and wrote the manuscript with input from all authors.

DECLARATION OF INTERESTS

C.K., T.T., A.K., Y.P., P.S., L.C., W.R., S.M., S.N., M.D., R.I., S.Y., P.McQ., C.T.W., and T.G.L. were employees of Takeda Pharmaceutical Company Inc. and had ownership of Takeda stock during the execution of the reported studies. W.R. and R.I. are current employees of Crosswalk Therapeutics and may have stock ownership. M.D. is a current employee of Dyno Therapeutics and may have stock ownership. The study was funded by Takeda Pharmaceutical Company Inc. A US patent application titled "PET Trace Compounds and Reaction Intermediates, and Method of Use"; USPTO Application no. 63/502,761″ was submitted. C.C.C. and A.L.A. were employees of Invicro. S.H.A., A.P.B., L.M.C., and Q.-D.N. have declared that no competing interest exists.

SUPPLEMENTAL INFORMATION

Supplemental information can be found online at https://doi.org/10.1016/j.ymthe.2024.11.021.

REFERENCES

1. Baig, S., Edward, N.C., Kotecha, D., Liu, B., Nordin, S., Kozor, R., Moon, J.C., Geberhiwot, T., and Steeds, R.P. (2018). Ventricular arrhythmia and sudden cardiac death in Fabry disease: a systematic review of risk factors in clinical practice. Europace. *20*, f153–f161. https://doi.org/10.1093/europace/eux261.

2. Azevedo, O., Gago, M.F., Miltenberger-Miltenyi, G., Sousa, N., and Cunha, D. (2020). Fabry Disease Therapy: State-of-the-Art and Current Challenges. Int. J. Mol. Sci. *22*, 206. https://doi.org/10.3390/ijms22010206.

3. Van Diggelen, O.P., Schram, A.W., Sinnott, M.L., Smith, P.J., Robinson, D., and Galjaard, H. (1981). Turnover of beta-galactosidase in fibroblasts from patients with genetically different types of beta-galactosidase deficiency. Biochem. J. *200*, 143–151. https://doi.org/10.1042/bj2000143.

4. Kaufmann, K.B., Büning, H., Galy, A., Schambach, A., and Grez, M. (2013). Gene therapy on the move. EMBO Mol. Med. *5*, 1642–1661. https://doi.org/10.1002/emmm.201202287.

5. Chen, N., Ehmann, D.E., Crooker, R., Derakhchan, K., Fang, X., Felice, B., Galbreath, E.J., Glaus, C., Gu, H., Huang, Y., et al. (2023). Gene therapy for cross-correction of somatic organs and the CNS in mucopolysaccharidosis II in rodents and non-human primates. Mol. Ther. Methods Clin. Dev. *29*, 286–302. https://doi.org/10.1016/j.omtm.2023.03.014.

6. Lohith, T.G., Furukawa, T., Mori, T., Kobayashi, M., and Fujibayashi, Y. (2008). Basic evaluation of FES-hERL PET tracer-reporter gene system for in vivo monitoring of adenoviral-mediated gene therapy. Mol. Imaging Biol. *10*, 245–252. https://doi.org/10.1007/s11307-008-0149-0.

7. Shah, K., Jacobs, A., Breakefield, X.O., and Weissleder, R. (2004). Molecular imaging of gene therapy for cancer. Gene Ther. *11*, 1175–1187. https://doi.org/10.1038/sj.gt.3302278.

8. Lohith, T.G., Kaittanis, C., Belanger, A.P., Ahn, S.H., Sandoval, P., Cohen, L., Rajarshi, G., Ruangsiriluk, W., Islam, R., Winkelmann, C.T., and McQuade, P. (2023). Radiosynthesis and Early Evaluation of a Positron Emission Tomography Imaging Probe [^{18}F]AGAL Targeting Alpha-Galactosidase A Enzyme for Fabry Disease. Molecules *28*, 7144. https://doi.org/10.3390/molecules28207144.

9. Willems, L.I., Beenakker, T.J.M., Murray, B., Scheij, S., Kallemeijn, W.W., Boot, R.G., Verhoek, M., Donker-Koopman, W.E., Ferraz, M.J., van Rijssel, E.R., et al. (2014). Potent and selective activity-based probes for GH27 human retaining alpha-galactosidases. J. Am. Chem. Soc. *136*, 11622–11625. https://doi.org/10.1021/ja507040n.

10. Chanda, D., Hensel, J.A., Higgs, J.T., Grover, R., Kaza, N., and Ponnazhagan, S. (2017). Effects of Cellular Methylation on Transgene Expression and Site-Specific Integration of Adeno-Associated Virus. Genes (Basel) *8*, 232. https://doi.org/10.3390/genes8090232.

11. McCafferty, E.H., and Scott, L.J. (2019). Migalastat: A Review in Fabry Disease. Drugs *79*, 543–554. https://doi.org/10.1007/s40265-019-01090-4.

12. Wu, Y.S., Khanna, R., Schmith, V., Lun, Y., Shen, J.S., Garcia, A., Dungan, L., Perry, A., Martin, L., Tsai, P.C., et al. (2021). Migalastat Tissue Distribution: Extrapolation From Mice to Humans Using Pharmacokinetic Modeling and Comparison With Agalsidase Beta Tissue Distribution in Mice. Clin. Pharmacol. Drug Dev. *10*, 1075–1088. https://doi.org/10.1002/cpdd.941.

13. Lohith, T.G., Zoghbi, S.S., Morse, C.L., Araneta, M.D.F., Barth, V.N., Goebl, N.A., Tauscher, J.T., Pike, V.W., Innis, R.B., and Fujita, M. (2014). Retest imaging of [11C]NOP-1A binding to nociceptin/orphanin FQ peptide (NOP) receptors in the brain of healthy humans. Neuroimage *87*, 89–95. https://doi.org/10.1016/j.neuroimage.2013.10.068.

14. Zanotti-Fregonara, P., Lammertsma, A.A., and Innis, R.B. (2013). Suggested pathway to assess radiation safety of [^{18}F]-labeled PET tracers for first-in-human studies. Eur. J. Nucl. Med. Mol. Imaging *40*, 1781–1783. https://doi.org/10.1007/s00259-013-2512-x.

15. Colella, P., Ronzitti, G., and Mingozzi, F. (2018). Emerging Issues in AAV-Mediated *In Vivo* Gene Therapy. Mol. Ther. Methods Clin. Dev. *8*, 87–104. https://doi.org/10.1016/j.omtm.2017.11.007.

16. Germain, D.P., Fouilhoux, A., Decramer, S., Tardieu, M., Pillet, P., Fila, M., Rivera, S., Deschênes, G., and Lacombe, D. (2019). Consensus recommendations for diagnosis, management and treatment of Fabry disease in paediatric patients. Clin. Genet. *96*, 107–117. https://doi.org/10.1111/cge.13546.

17. Germain, D.P., Elliott, P.M., Falissard, B., Fomin, V.V., Hilz, M.J., Jovanovic, A., Kantola, I., Linhart, A., Mignani, R., Namdar, M., et al. (2019). The effect of enzyme replacement therapy on clinical outcomes in male patients with Fabry disease: A systematic literature review by a European panel of experts. Mol. Genet. Metab. Rep. *19*, 100454. https://doi.org/10.1016/j.ymgmr.2019.100454.

18. Touboul, D., Roy, S., Germain, D.P., Baillet, A., Brion, F., Prognon, P., Chaminade, P., and Laprévote, O. (2005). Fast fingerprinting by MALDI-TOF mass spectrometry of urinary sediment glycosphingolipids in Fabry disease. Anal. Bioanal. Chem. *382*, 1209–1216. https://doi.org/10.1007/s00216-005-3239-8.

19. Maruyama, H., Miyata, K., Mikame, M., Taguchi, A., Guili, C., Shimura, M., Murayama, K., Inoue, T., Yamamoto, S., Sugimura, K., et al. (2019). Effectiveness of plasma lyso-Gb3 as a biomarker for selecting high-risk patients with Fabry disease from multispecialty clinics for genetic analysis. Genet. Med. *21*, 44–52. https://doi.org/10.1038/gim.2018.31.

20. Nowak, A., Mechtler, T.P., Desnick, R.J., and Kasper, D.C. (2017). Plasma LysoGb3: A useful biomarker for the diagnosis and treatment of Fabry disease heterozygotes. Mol. Genet. Metab. *120*, 57–61. https://doi.org/10.1016/j.ymgme.2016.10.006.

21. Sakuraba, H., Togawa, T., Tsukimura, T., and Kato, H. (2018). Plasma lyso-Gb3: a biomarker for monitoring fabry patients during enzyme replacement therapy. Clin. Exp. Nephrol. *22*, 843–849. https://doi.org/10.1007/s10157-017-1525-3.

22. Spada, M., Baron, R., Elliott, P.M., Falissard, B., Hilz, M.J., Monserrat, L., Tøndel, C., Tylki-Szymańska, A., Wanner, C., and Germain, D.P. (2019). The effect of enzyme replacement therapy on clinical outcomes in paediatric patients with Fabry disease - A systematic literature review by a European panel of experts. Mol. Genet. Metab. *126*, 212–223. https://doi.org/10.1016/j.ymgme.2018.04.007.

23. Najafian, B., Mauer, M., Hopkin, R.J., and Svarstad, E. (2013). Renal complications of Fabry disease in children. Pediatr. Nephrol. *28*, 679–687. https://doi.org/10.1007/s00467-012-2222-9.

24. Meyer, J.P., Adumeau, P., Lewis, J.S., and Zeglis, B.M. (2016). Click Chemistry and Radiochemistry: The First 10 Years. Bioconjug. Chem. *27*, 2791–2807. https://doi.org/10.1021/acs.bioconjchem.6b00561.

25. Keu, K.V., Witney, T.H., Yaghoubi, S., Rosenberg, J., Kurien, A., Magnusson, R., Williams, J., Habte, F., Wagner, J.R., Forman, S., et al. (2017). Reporter gene imaging of targeted T cell immunotherapy in recurrent glioma. Sci. Transl. Med. *9*, eaag2196. https://doi.org/10.1126/scitranslmed.aag2196.

26. Pearson, T.S., Gupta, N., San Sebastian, W., Imamura-Ching, J., Viehoever, A., Grijalvo-Perez, A., Fay, A.J., Seth, N., Lundy, S.M., Seo, Y., et al. (2021). Gene therapy for aromatic L-amino acid decarboxylase deficiency by MR-guided direct delivery of AAV2-AADC to midbrain dopaminergic neurons. Nat. Commun. *12*, 4251. https://doi.org/10.1038/s41467-021-24524-8.

27. Shaikh, F.A., Kurtys, E., Kubassova, O., and Roettger, D. (2020). Reporter gene imaging and its role in imaging-based drug development. Drug Discov. Today *25*, 582–592. https://doi.org/10.1016/j.drudis.2019.12.010.

28. Yaghoubi, S.S., Campbell, D.O., Radu, C.G., and Czernin, J. (2012). Positron emission tomography reporter genes and reporter probes: gene and cell therapy applications. Theranostics *2*, 374–391. https://doi.org/10.7150/thno.3677.

29. Sellmyer, M.A., Richman, S.A., Lohith, K., Hou, C., Weng, C.C., Mach, R.H., O'Connor, R.S., Milone, M.C., and Farwell, M.D. (2020). Imaging CAR T Cell Trafficking with eDHFR as a PET Reporter Gene. Mol. Ther. *28*, 42–51. https://doi.org/10.1016/j.ymthe.2019.10.007.

30. Tai, C.H., Lee, N.C., Chien, Y.H., Byrne, B.J., Muramatsu, S.I., Tseng, S.H., and Hwu, W.L. (2022). Long-term efficacy and safety of eladocagene exuparvovec in patients with AADC deficiency. Mol. Ther. *30*, 509–518. https://doi.org/10.1016/j.ymthe.2021.11.005.

31. Umer, M., and Kalra, D.K. (2023). Treatment of Fabry Disease: Established and Emerging Therapies. Pharmaceuticals (Basel) *16*, 320. https://doi.org/10.3390/ph16020320.

32. Gaertner, F.C., Fürst, S., and Schwaiger, M. (2013). PET/MR: a paradigm shift. Cancer Imaging *13*, 36–52. https://doi.org/10.1102/1470-7330.2013.0005.

33. Katal, S., Eibschutz, L.S., Saboury, B., Gholamrezanezhad, A., and Alavi, A. (2022). Advantages and Applications of Total-Body PET Scanning. Diagnostics (Basel) *12*, 426. https://doi.org/10.3390/diagnostics12020426.

34. Alcaide, A., Trapero, A., Pérez, Y., and Llebaria, A. (2015). Galacto configured N-aminoaziridines: a new type of irreversible inhibitor of beta-galactosidases. Org. Biomol. Chem. *13*, 5690–5697. https://doi.org/10.1039/c5ob00532a.

35. Hansen, F.G., Bundgaard, E., and Madsen, R. (2005). A short synthesis of (+)-cyclophellitol. J. Org. Chem. *70*, 10139–10142. https://doi.org/10.1021/jo051645q.

36. Harrak, Y., Barra, C.M., Delgado, A., Castaño, A.R., and Llebaria, A. (2011). Galacto-configured aminocyclitol phytoceramides are potent in vivo invariant natural killer

T cell stimulators. J. Am. Chem. Soc. *133*, 12079–12084. https://doi.org/10.1021/ja202610x.

37. Boukharov, N., Yuan, S., Ruangsiriluk, W., Ayyadurai, S., Rahman, A., Rivera-Hernandez, M., Sunkara, S., Tonini, K., Park, E.Y., Deshpande, M., and Islam, R. (2024). Developing gene therapy for mitigating multisystemic pathology in Fabry disease: proof of concept in an aggravated mouse model. Hum. Gene Ther. *35*, 680–694. https://doi.org/10.1089/hum.2023.222.

38. Knight, A.C., Varlow, C., Zi, T., Liang, S.H., Josephson, L., Schmidt, K., Patel, S., and Vasdev, N. (2021). In Vitro Evaluation of [^{3}H]CPPC as a Tool Radioligand for CSF-1R. ACS Chem. Neurosci. *12*, 998–1006. https://doi.org/10.1021/acschemneuro.0c00802.

YMTHE, Volume *33*

Supplemental Information

Longitudinal imaging of therapeutic enzyme expression after gene therapy for Fabry disease using positron emission tomography and the radiotracer [^{18}F]AGAL

Charalambos Kaittanis, Tyler Teceno, Ashley Knight, Yoann Petibon, Phil Sandoval, Lawrence Cohen, Shin Hye Ahn, Anthony P. Belanger, Louise M. Clark, Quang-De Nguyen, Wanida Ruangsiriluk, Shreya Mukherji, Cristian C. Constantinescu, Amy Llopis Amenta, Sarav Narayanan, Mugdha Deshpande, Rizwana Islam, Shipeng Yuan, Paul McQuade, Christopher T. Winkelmann, and Talakad G. Lohith

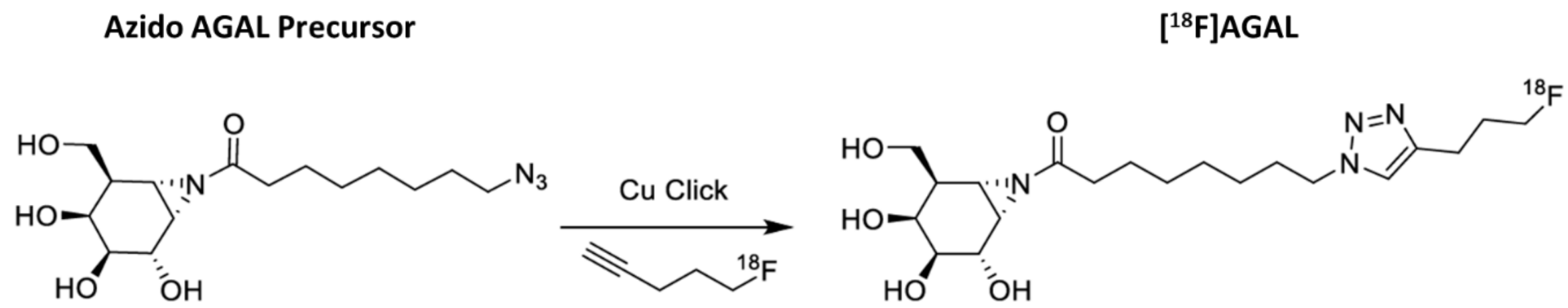

Figure S1: Radiolabeling of the azide-containing AGAL precursor with Fluorine-18, to yield the PET tracer [^{18}F]AGAL after Cu(I)-catalyzed azide–alkyne 1,3-dipolar cycloaddition (CuAAC; click chemistry).

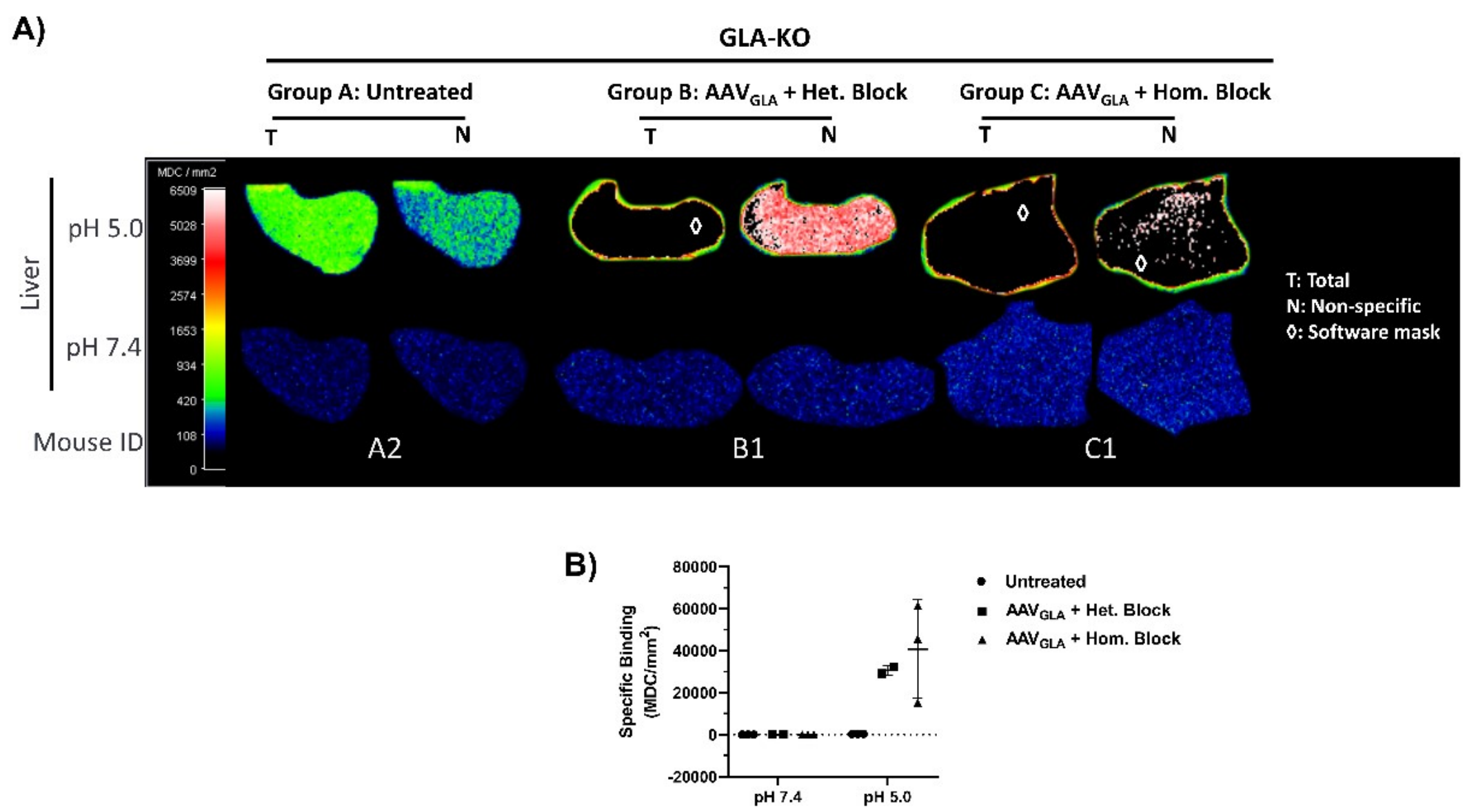

Figure S2. ***In vitro*** **characterization of the [^{18}F]AGAL probe demonstrating binding to human alpha galactosidase A in the liver of transduced Fabry (GLA KO) mice. A:** Representative autoradiography images of mouse liver tissue sections incubated with [^{18}F]AGAL at the corresponding pH, indicating that the probe binds to the enzyme in acidic conditions (pH 5.0). **B:** Quantification of the autoradiography analysis, demonstrating increased specific binding of [^{18}F]AGAL to the livers of Fabry mice transduced with rAAV-ubp-α-GalA. Data are displayed as means and standard deviations with illustration of individual datapoints. (MDC/mm^2: molecular dynamic counts per square millimeter; GLA-KO: GLA knockout Fabry mice; rAAV-ubp-α-GalA: AAV encoding human alpha galactosidase A; Het. and Hom. Block: heterologous and homologous blocking respectively; Software-masked area represents part of the slice where the counts reached saturation).

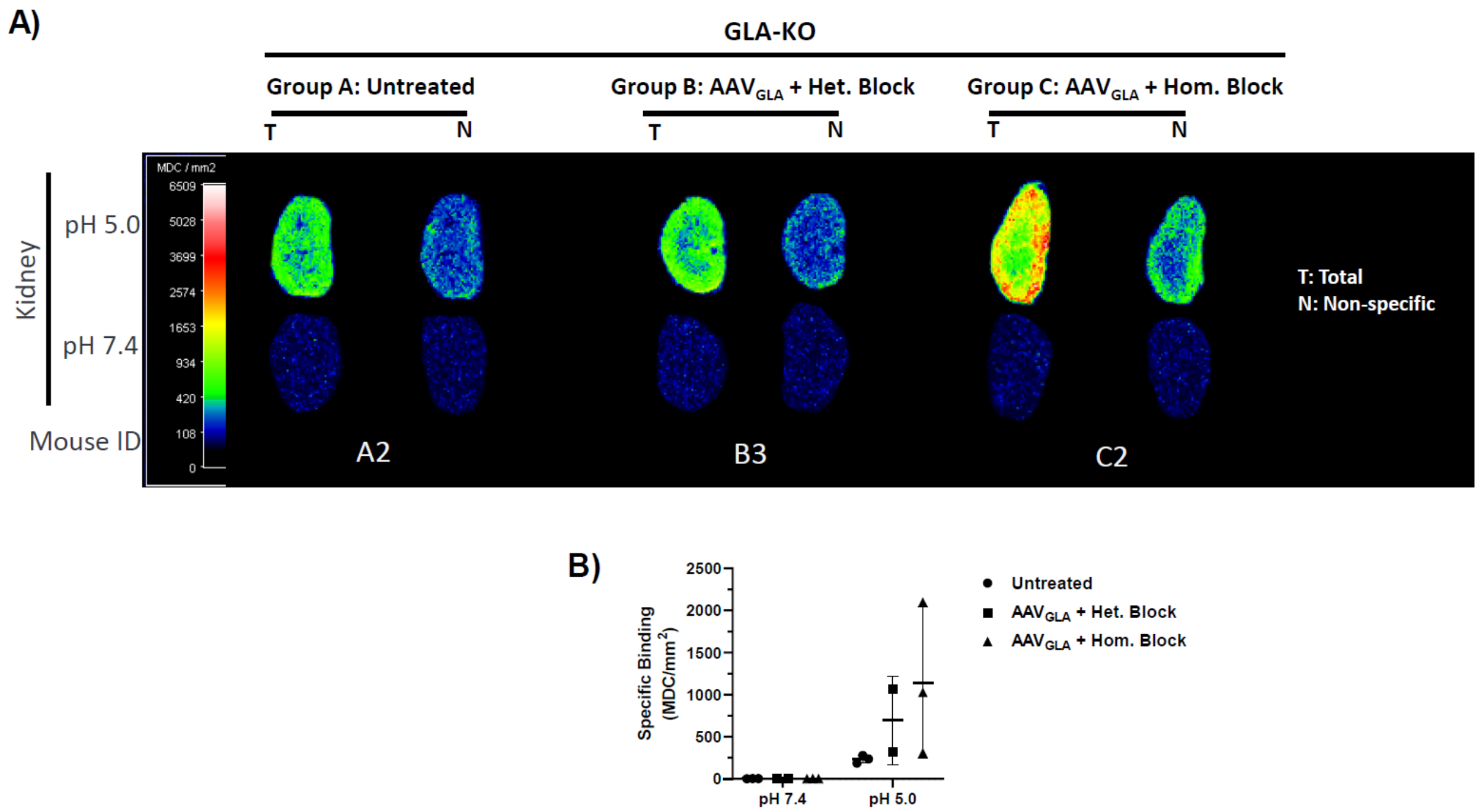

Figure S3. ***In vitro*** **characterization of the [^{18}F]AGAL probe demonstrating binding to human alpha galactosidase A in the kidney of transduced Fabry (GLA KO) mice. A:** Representative autoradiography images of mouse kidney tissue sections incubated with [^{18}F]AGAL at the corresponding pH, indicating that the probe binds to the enzyme in acidic conditions (pH 5.0). **B:** Quantification of the autoradiography analysis, demonstrating increased specific binding of [^{18}F]AGAL to the kidneys of Fabry mice transduced with rAAV-ubp-α-GalA. Data are displayed as means and standard deviations with illustration of individual datapoints. (MDC/mm^2: molecular dynamic counts per square millimeter; GLA-KO: GLA knockout Fabry mice; rAAV-ubp-α-GalA: AAV encoding human alpha galactosidase A; Het. and Hom. Block: heterologous and homologous blocking respectively).

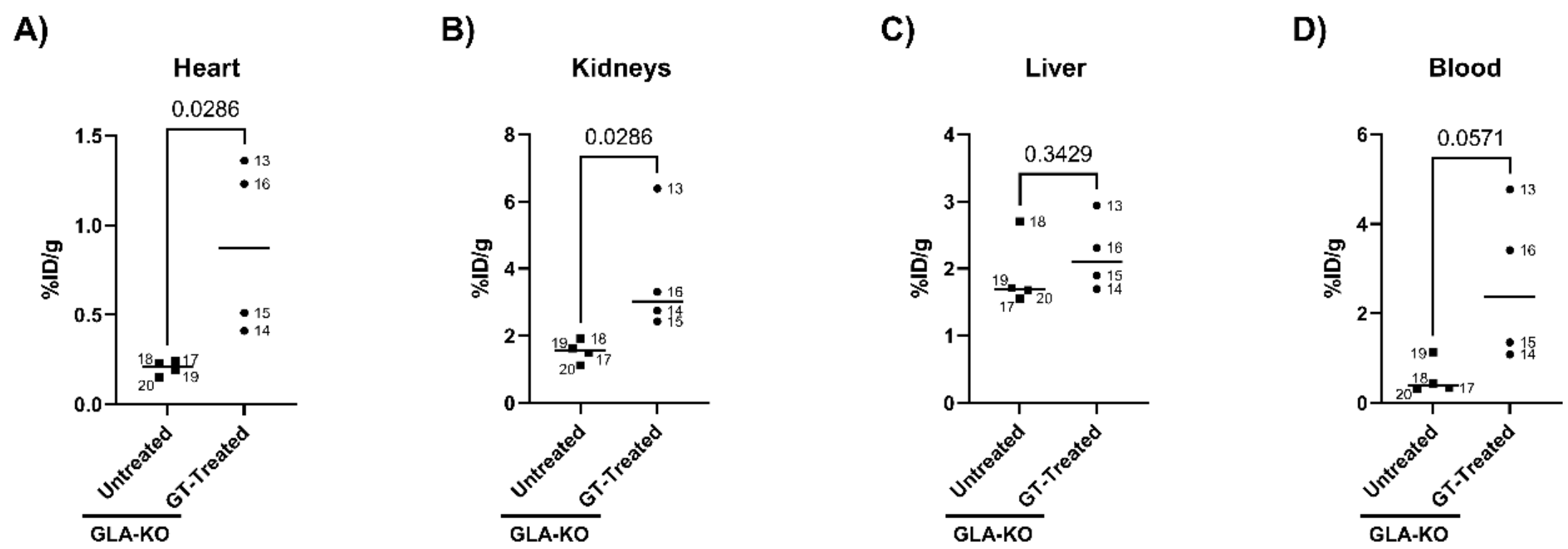

Figure S4. ***Ex vivo*** **evaluation of the uptake of the [^{18}F]AGAL probe in Fabry (GLA KO) mice.** Quantification of the tracer's levels in the **(A)** heart, **(B)** kidneys, **(C)** liver, and **(D)** blood using a gamma counter. Data are displayed as means with illustration of individual datapoints. The animals' identifiers are next to the corresponding datapoint. Statistical significance was determined using the Mann-Whitney test, and the p value is stated on top of the horizonal bar that compares the two groups. (%ID/g: percent injected dose of [^{18}F]AGAL per gram of tissue/blood; GLA-KO: GLA knockout Fabry mice; GT-Treated: animals transduced with AAV encoding human alpha galactosidase A).

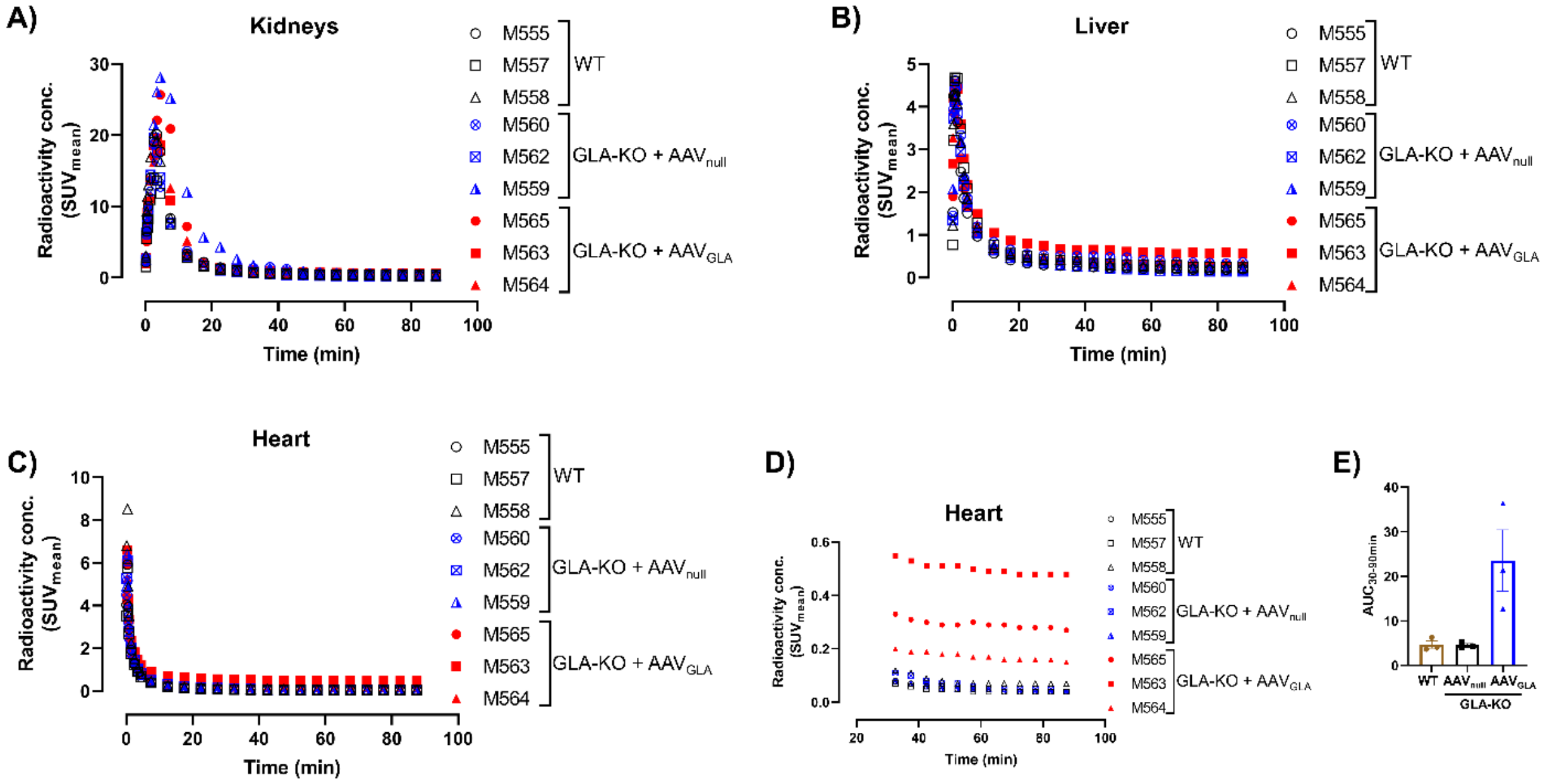

Figure S5. Time-activity uptake curves of major organs in GLA-KO mice 6 weeks after receiving gene therapy. A-C: PET-based determination of the kinetics of [^{18}F]AGAL in kidneys, liver, and heart. The animals' identifiers are depicted in the plots. **D:** [^{18}F]AGAL uptake in the heart from 30to 90 minutes, showing higher retention in the AAV_{GLA}-treated mice. **E:** Fabry mice that underwent gene therapy that encodes human alpha galactosidase A have higher [^{18}F]AGAL uptake in their hearts. Data are displayed as means and standard deviations with illustration of individual datapoints. (SUV_{mean}: mean standardized uptake value; WT: wildtype; GLA-KO: GLA knockout Fabry mice; AAV_{GLA}: AAV encoding human alpha galactosidase A; AAV_{null}: AAV not encoding a therapeutic protein; AUC: area-under-curve).

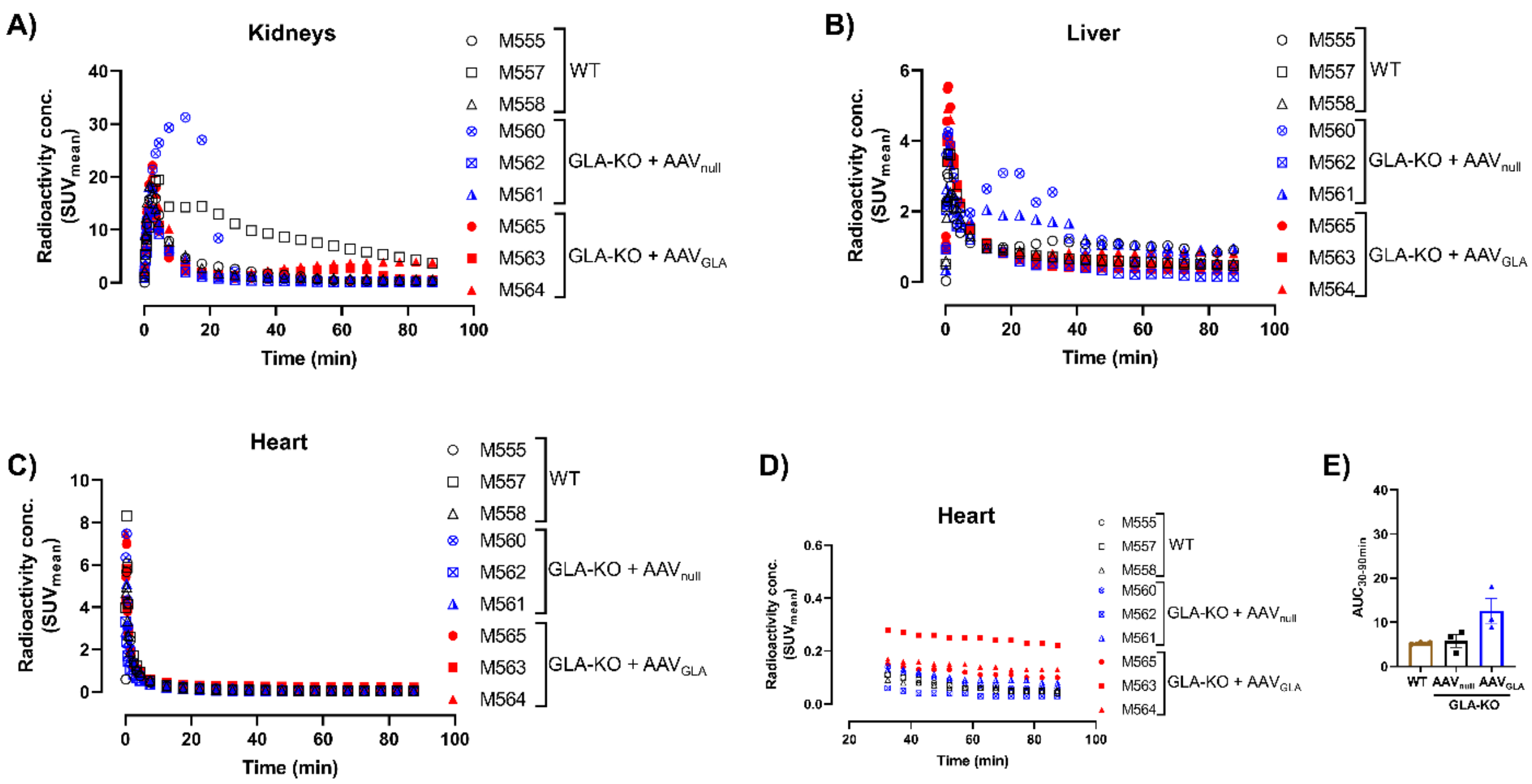

Figure S6. Time-activity uptake curves of major in GLA-KO mice 26 weeks after receiving gene therapy. A-C: PET-based determination of the kinetics of [^{18}F]AGAL in kidneys, liver, and heart. The animals' identifiers are depicted in the plots. **D:** [^{18}F]AGAL uptake in the heart from 30 to 90 minutes, showing higher retention in the AAV_{GLA}-treated mice. **E:** Fabry mice that underwent gene therapy that encodes human alpha galactosidase A have higher [^{18}F]AGAL uptake in their hearts. Data are displayed as means and standard deviations with illustration of individual datapoints. (SUV_{mean}: mean standardized uptake value; WT: wildtype; GLA-KO: GLA knockout Fabry mice; AAV_{GLA}: AAV encoding human alpha galactosidase A; AAV_{null}: AAV not encoding a therapeutic protein; AUC: area-under-curve).

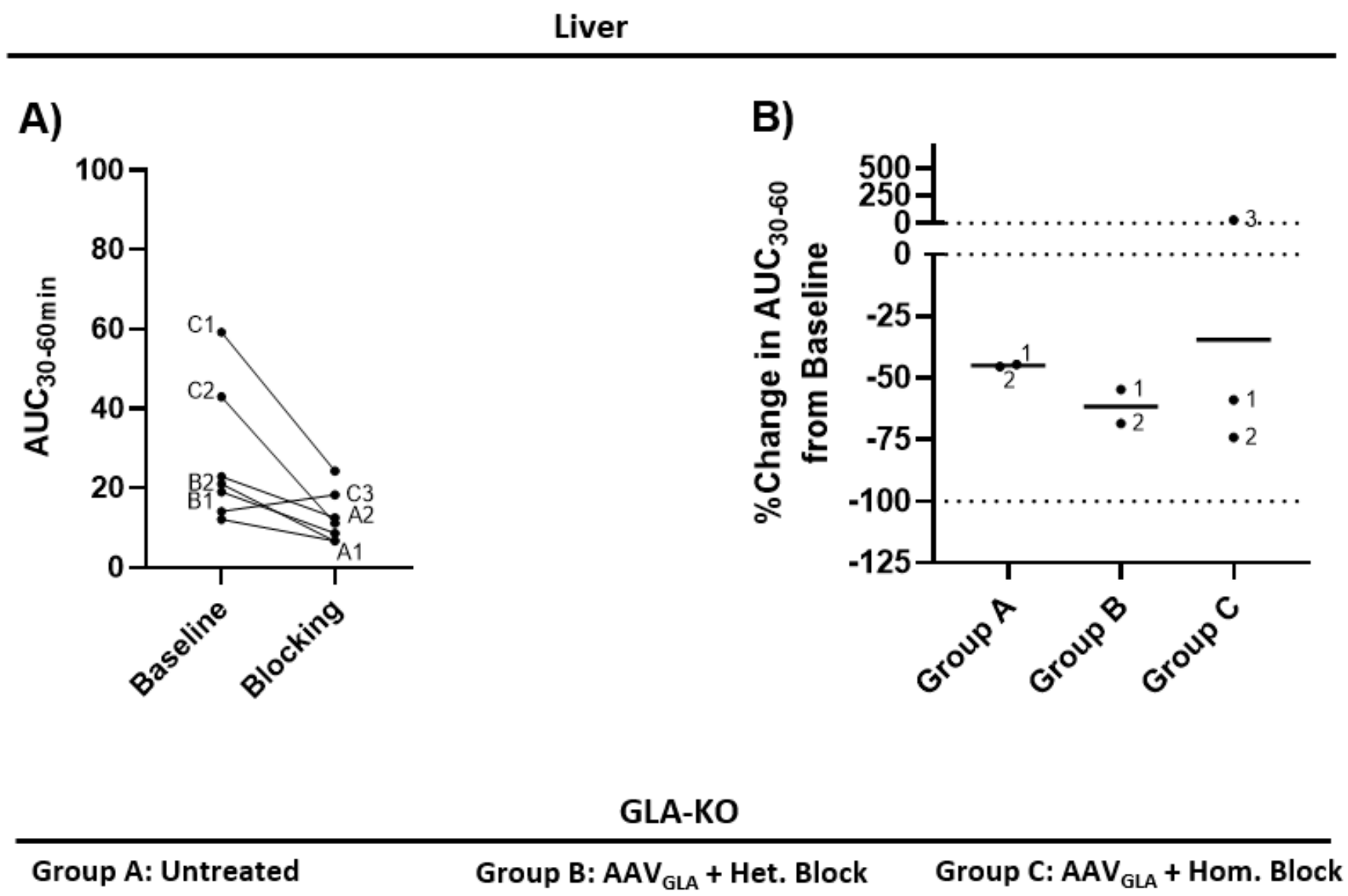

Figure S7. *In vivo* determination of the specificity of [^{18}F]AGAL binding in the liver of gene-therapy-treated Fabry (GLA-KO) mice. A: Quantification of the PET signal in the liver, and **(B)** determination of the changes in the tracer's uptake due to the blocking agent. Each datapoint depicts the corresponding animal identifiers. Each group's horizontal bar in panel **(B)** depicts the group mean. Heterologous block was done with Migalastat, and homologous block was achieved with [^{19}F]AGAL (Migalastat and [^{19}F]AGAL were dosed at 1 mg/kg). (GLA KO: GLA knockout Fabry mice; AUC_{30-60}: area-under-curve of 30 to 60 minutes post tracer dosing; AAV_{GLA}: AAV encoding human alpha galactosidase A; Het. and Hom. Block: heterologous and homologous blocking respectively).

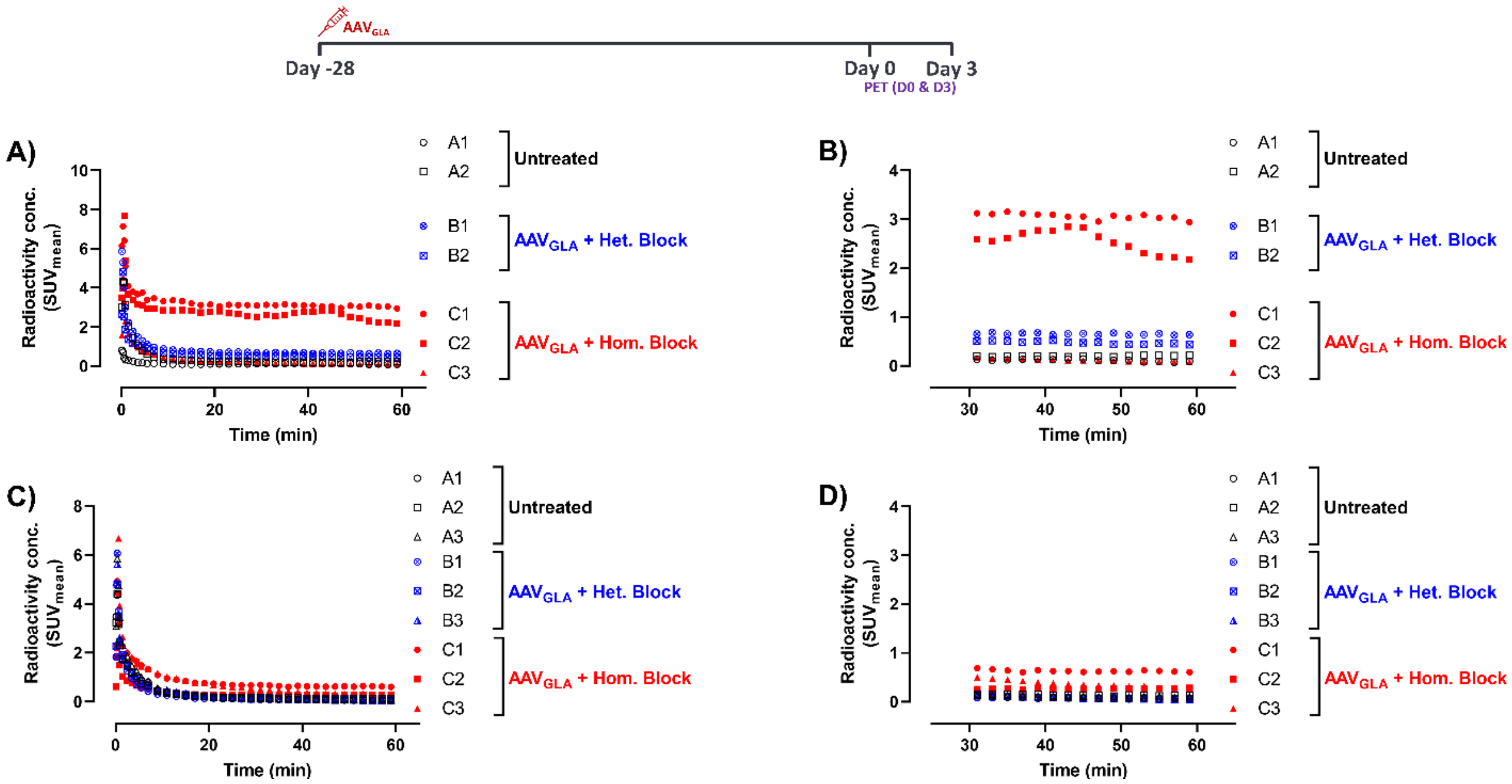

Figure S8. Heart time-activity curves in GLA-KO mice after receiving gene therapy, where [18F]AGAL was administered after the blocking agent. PET-based determination of the kinetics of [18F]AGAL in the heart **(A-B)** on Imaging Day 0 (28 days after dosing of AAV_{GLA} as baseline) and **(C-D)** on Imaging Day 3 (following administration of a heterologous or homologous blocking agent. Heterologous block was done with Migalastat, and homologous block was achieved with [19F]AGAL (Migalastat and [19F]AGAL were dosed at 1 mg/kg). Reduction in the uptake of the tracer due to blocking is observed on Imaging Day 3. The animals' identifiers are depicted in the plots. (SUV_{mean}: mean standardized uptake value; GLA-KO: GLA knockout Fabry mice; AAV_{GLA}: AAV encoding human alpha galactosidase A; Het. and Hom. Block: heterologous and homologous blocking respectively).

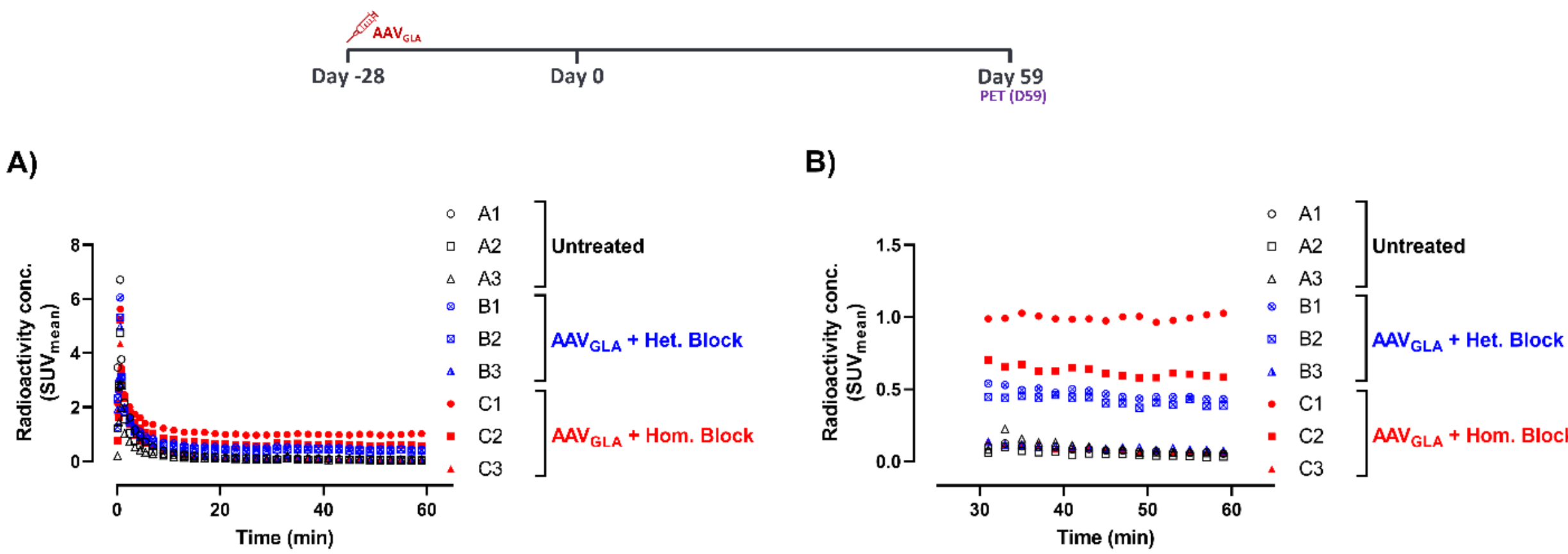

Figure S9. Heart time-activity curves in GLA-KO mice after receiving gene therapy, where [^{18}F]AGAL was administered 30 minutes before the blocking agent. PET-based determination of the kinetics of [^{18}F]AGAL in the heart **(A-B)** on Imaging Day 59 (59 days after dosing of AAV_{GLA} as baseline). Heterologous block was done with Migalastat, and homologous block was achieved with [^{19}F]AGAL (Migalastat and [^{19}F]AGAL were dosed at 1 mg/kg). AAV_{GLA}-treated mice maintained high tracer uptake, suggesting that the tracer cannot be displaced by the blocking compound. The animals' identifiers are depicted in the plots. (SUV_{mean}: mean standardized uptake value; GLA-KO: GLA knockout Fabry mice; AAV_{GLA}: AAV encoding human alpha galactosidase A; Het. and Hom. Block: heterologous and homologous blocking respectively).

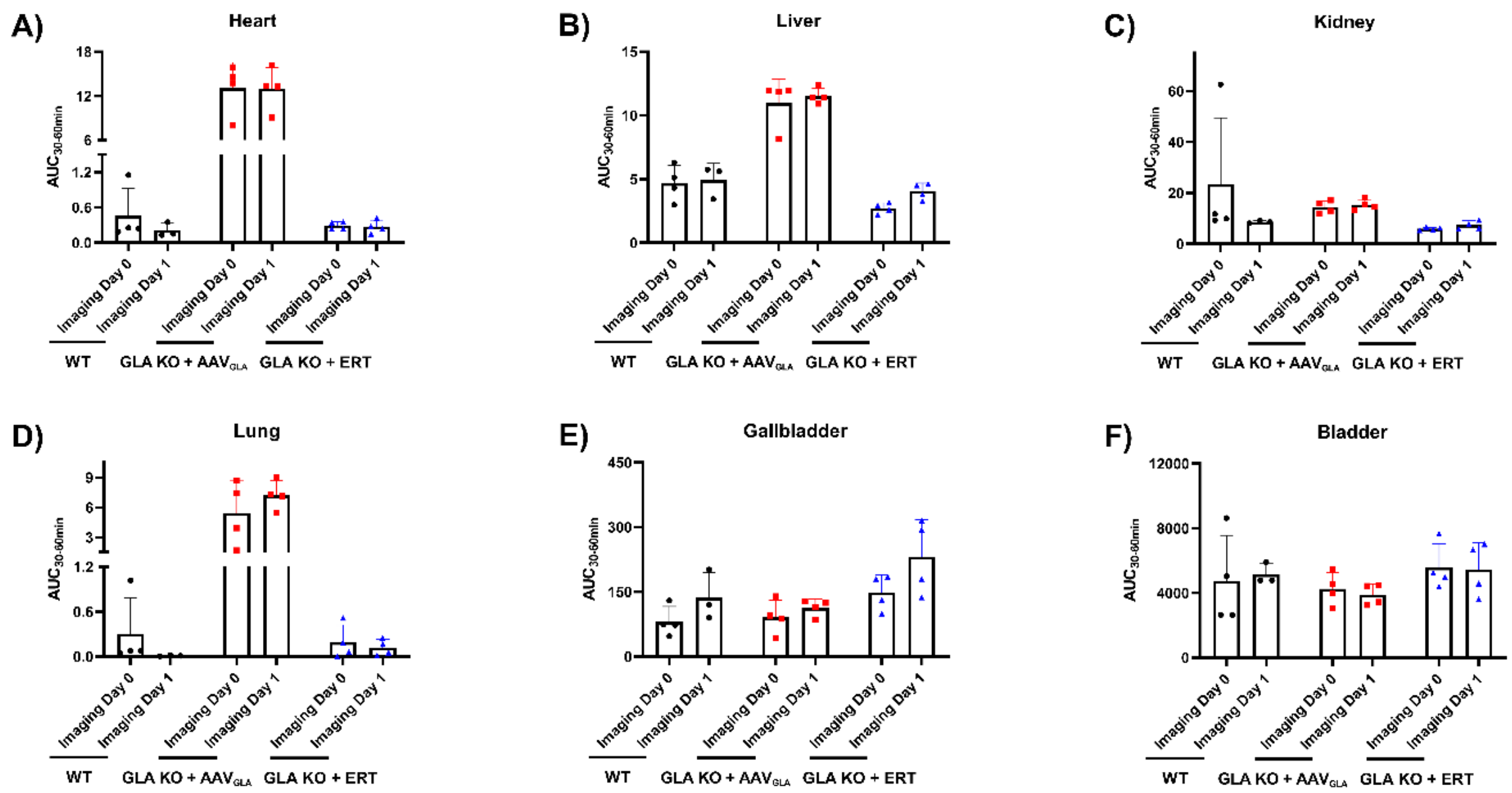

Figure S10. ***In vivo*** **comparison of [^{18}F]AGAL uptake in gene-therapy-** ***vs*** **enzyme-replacement-treated Fabry (GLA KO) mice.** PET-based determination of the retention of [^{18}F]AGAL in **(A)** the heart, **(B)** liver, **(C)** kidney, **(D)** lung, **(E)** gallbladder, and **(F)** bladder. Higher uptake is observed in the heart, liver, and lung of gene-therapy-treated animals. Data are displayed as means and standard deviations. (AUC_{30-60}: area-under-curve of 30 to 60 minutes post tracer dosing; WT: wildtype mice; GLA-KO: GLA knockout Fabry mice; AAV_{GLA}: AAV encoding human alpha galactosidase A; ERT: enzyme replacement therapy).

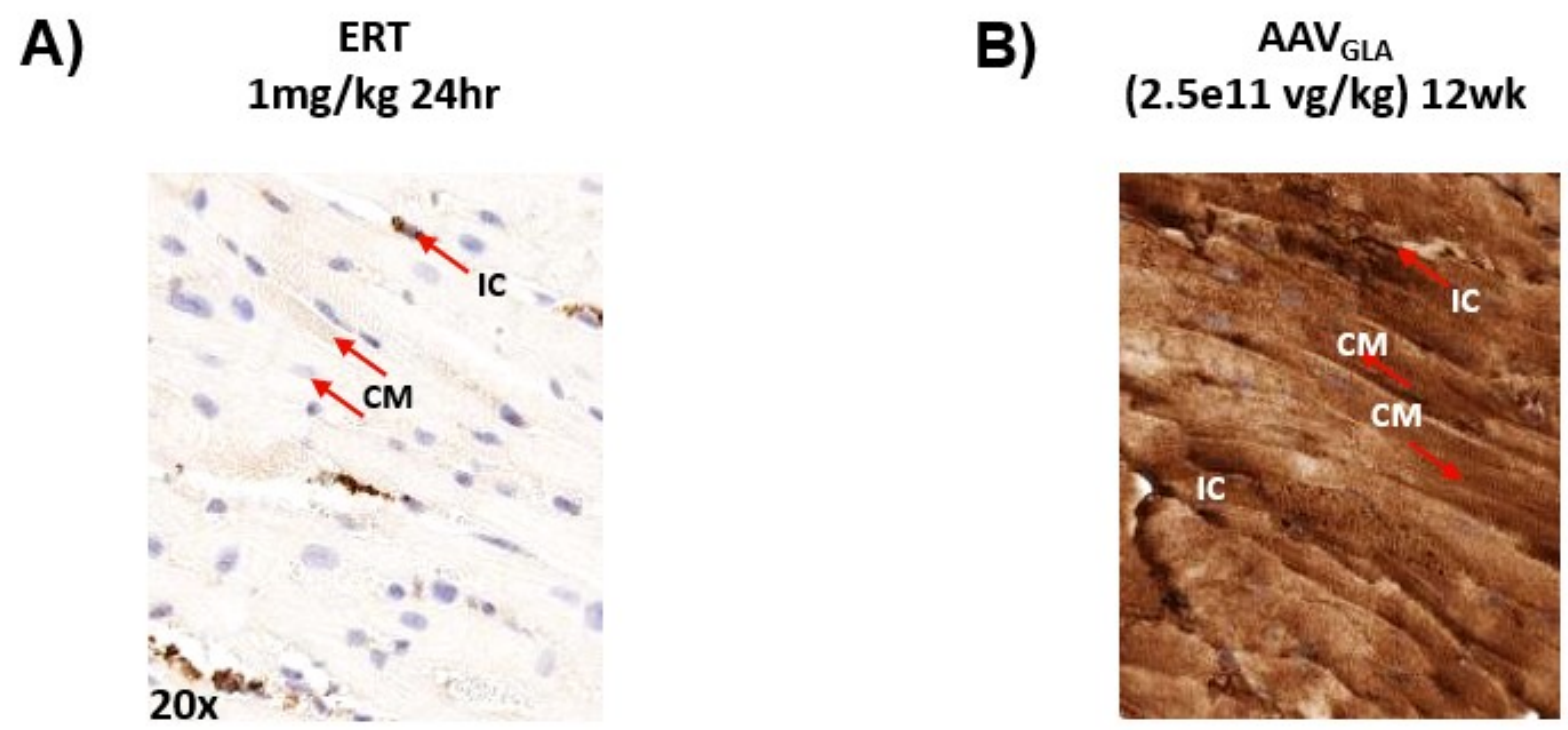

Figure S11. Immunohistochemical evaluation of the heart of Fabry (GLA KO) mice for human alpha galactosidase A. Representative immunohistochemistry images of heart tissue sections of animals that didn't undergo PET imaging are shown. **A:** alpha galactosidase A is detected 24 hours after enzyme replacement therapy (ERT). **B:** The enzyme is detected in multiple cells 12 weeks after gene therapy, with pronounced staining observed in the mice that received AAV_{GLA}. (AAV_{GLA}: AAV encoding human alpha galactosidase A; CM: cardiomyocytes; IC: interstitial cells; α-Gal A positive staining (brown)).

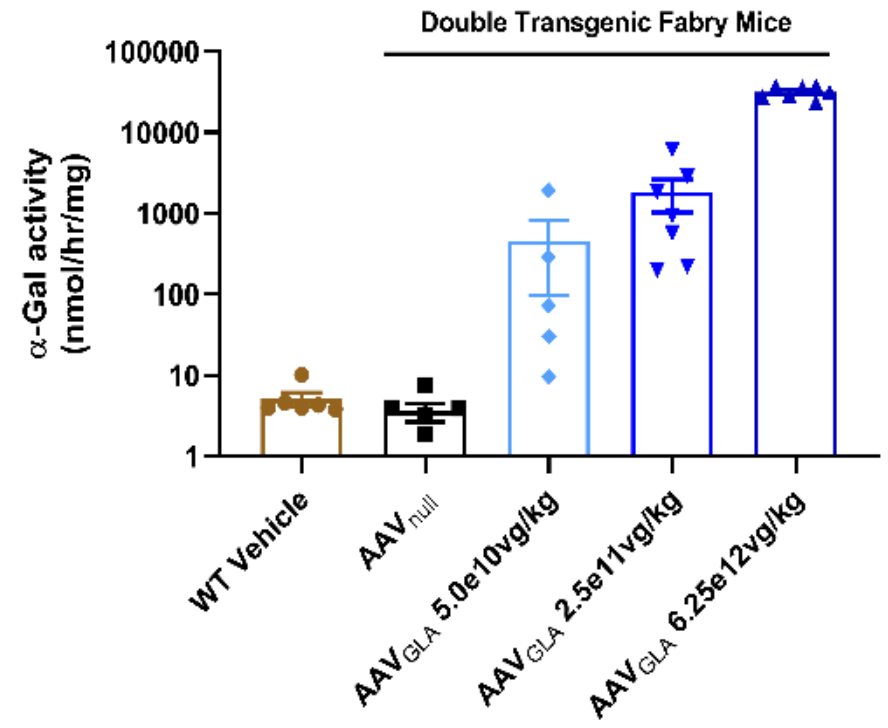

Figure S12. GLA activity in the heart after transduction with different AAV_{GLA} titers. Gene therapy of double transgenic Fabry mice (G3Stg/GLA KO) induces dose-dependent expression of active alpha galactosidase A in the heart. Data are displayed as means and standard deviations with illustration of individual datapoints on panel. (WT: wildtype mice; AAV_{GLA}: AAV encoding human alpha galactosidase A; AAV_{null}: AAV not encoding a therapeutic protein; vg/kg: viral genome copies per kilogram).

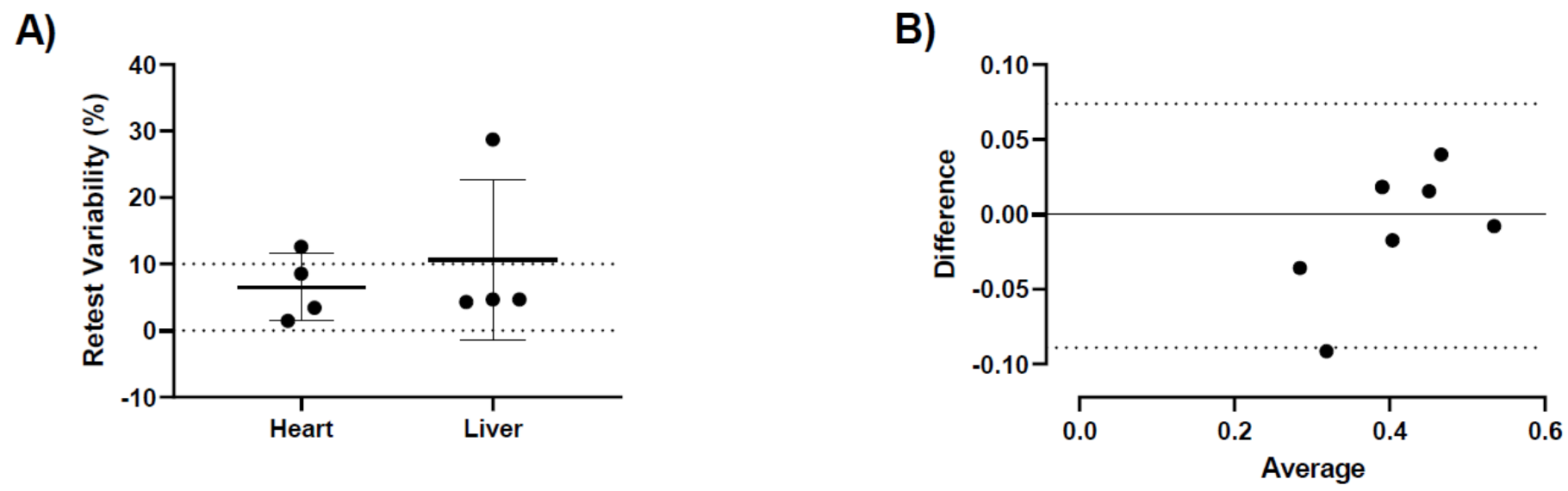

Figure S13. Retest variability and Bland-Altman plot of [^{18}F]AGAL. A: The tracer's retest variability in the heart and liver was determined using AAV_{GLA}-treated Fabry mice that were imaged on two consecutive days (Imaging Day 0 and 1). Tracers with retest variability under 10 % are considered excellent. **B:** In the Bland-Altman plot the solid middle line denotes the bias from zero, whereas the dashed lines depict the 95 % limits of agreement (i.e. bias ± 1.96 × standard deviation). Data are displayed as means and standard deviations with illustration of individual datapoints on panel A. (SUV: standardized uptake value; AAV_{GLA}: AAV encoding human alpha galactosidase A).

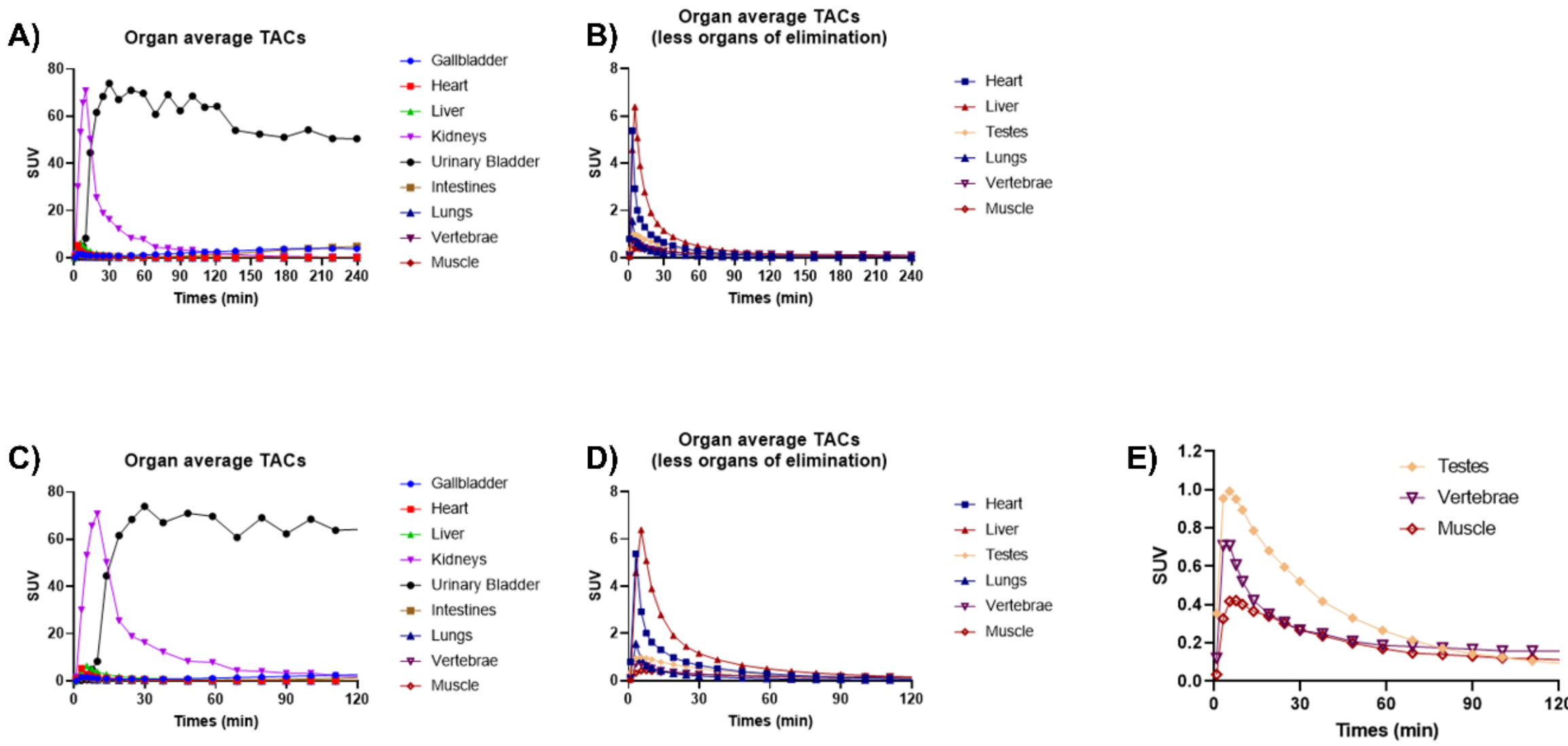

Figure S14. Time-activity uptake curves of [^{18}F]AGAL in male rhesus monkey (*Macaca mulatta*). PET-based determination of the kinetics of [^{18}F]AGAL in select organs following administration of the tracer. Panels **(A-B)** depict the kinetics for the duration of the scan, whereas panes **(C-E)** show [^{18}F]AGAL's kinetics up to 2 hours after intravenous administration. (SUV: standardized uptake value; TACs: time-activity curves).
